# Membrane perturbing properties of toxin mycolactone from *Mycobacterium ulcerans*

**DOI:** 10.1371/journal.pcbi.1005972

**Published:** 2018-02-05

**Authors:** Cesar A. López, Clifford J. Unkefer, Basil I. Swanson, Jessica M. J. Swanson, S. Gnanakaran

**Affiliations:** 1 Theoretical Biology and Biophysics Group, Los Alamos National Laboratory, New Mexico, United States of America; 2 Center for Nonlinear Studies, Los Alamos National Laboratory, New Mexico, United States of America; 3 BioSciences Division, Los Alamos National Laboratory, New Mexico, United States of America; 4 Department of Chemistry, University of Chicago, Illinois, United States of America; Max Planck Institute for Biophysical Chemistry, GERMANY

## Abstract

Mycolactone is the exotoxin produced by *Mycobacterium ulcerans* and is the virulence factor behind the neglected tropical disease Buruli ulcer. The toxin has a broad spectrum of biological effects within the host organism, stemming from its interaction with at least two molecular targets and the inhibition of protein uptake into the endoplasmic reticulum. Although it has been shown that the toxin can passively permeate into host cells, it is clearly lipophilic. Association with lipid carriers would have substantial implications for the toxin’s distribution within a host organism, delivery to cellular targets, diagnostic susceptibility, and mechanisms of pathogenicity. Yet the toxin’s interactions with, and distribution in, lipids are unknown. Herein we have used coarse-grained molecular dynamics simulations, guided by all-atom simulations, to study the interaction of mycolactone with pure and mixed lipid membranes. Using established techniques, we calculated the toxin’s preferential localization, membrane translocation, and impact on membrane physical and dynamical properties. The computed water-octanol partition coefficient indicates that mycolactone prefers to be in an organic phase rather than in an aqueous environment. Our results show that in a solvated membrane environment the exotoxin mainly localizes in the water-membrane interface, with a preference for the glycerol moiety of lipids, consistent with the reported studies that found it in lipid extracts of the cell. The calculated association constant to the model membrane is similar to the reported association constant for Wiskott-Aldrich syndrome protein. Mycolactone is shown to modify the physical properties of membranes, lowering the transition temperature, compressibility modulus, and critical line tension at which pores can be stabilized. It also shows a tendency to behave as a linactant, a molecule that localizes at the boundary between different fluid lipid domains in membranes and promotes inter-mixing of domains. This property has implications for the toxin’s cellular access, T-cell immunosuppression, and therapeutic potential.

## Author summary

Mycolactone is a macrolide exotoxin secreted by *Mycobacterium ulcerans*, which causes a skin disease called Buruli ulcer, a neglected emerging disease. It is the third most common mycobacterial disease after tuberculosis and leprosy. Studies have shown how mycolactone plays a pivotal role in Buruli ulcer pathogenesis, and identified it as an attractive therapeutic target. This multifunctional cytotoxin exerts multiple local and global responses, including ulcerative, analgesic, and anti-inflammatory effects. Prompted by its lipid-like structure, we used extensive multi-resolution simulations to probe mycolactone’s interactions with model membranes. Our results suggest that mycolactone is sequestered in membranes where it alters several dynamical, physical, and mechanical properties. It also behaves as a linactant, localizing at the interface between lipid domains and decreasing the inter-domain line tension. Our results shed light on how mycolactone permeates host cell membranes and is distributed between lipid and aqueous environments. These findings have significant implications for the toxin’s distribution in the host environment and mechanisms of pathogenicity. Understanding the toxin’s distribution and mechanism of trafficking will have ramifications for targeted diagnostics, therapeutic approaches, and our understanding of Buruli ulcer pathogenesis.

## Introduction

Buruli ulcer (BU) is a cutaneous disease caused by *Mycobacterium ulcerans*. The presence of necrosis, which is accompanied by surprisingly little inflammation or pain, is considered the most characteristic clinical presentation of BU disease [1–3]. The macrolide exotoxin mycolactone, which is secreted by *M*. *ulcerans*, is thought to be the key virulence factor, playing the central role in the pathogenesis of BU [4,5]. The exotoxin’s causative effect was brought to light when it was shown that the symptoms of BU could be replicated by injection of mycolactone alone [6]. Mycolactones exist in multiple isomeric forms [7] and have been shown to act both *in vivo* and *in vitro* on various mammalian cell types, including fibroblasts [7–11], adipocytes [12], keratinocytes [13], myocytes [6,14], macrophages [15,16], and T cells [17,18]. At the cellular level, mycolactones induce apoptosis, cytoskeletal rearrangements, impaired cytokine production, and interference with cellular signaling [19].

Biophysical experiments have shown that the mycolactone hijacks the Wiskott-Aldrich syndrome proteins (WASP and N-WASP), binding to them with 100-fold higher affinity than their natural activator CDC42 [20]. This in turn activates actin branching, leading to defective cell adhesion, uncontrolled directional migration, and eventually to cell death [21]. Recent work has also shown that mycolactone inhibits the Sec61-dependent translocation of proteins into the endoplasmic reticulum, a process that likely explains the absence of immune responses and inflammation in BU [22,23]. Additional recent work has shown that mycolactone may exert an analgesic effect by inhibiting signaling by the angiotensin II type 2 receptor (AT2R); it inhibits AT2R and leads to decreased hyperpolarization in mouse neuronal cells [24]. Collectively, these effects suggest that mycolactone disrupts normal host function via multiple pathways, resulting a suite of consequences and range of cellular cytotoxicity.

**Fig 1:**
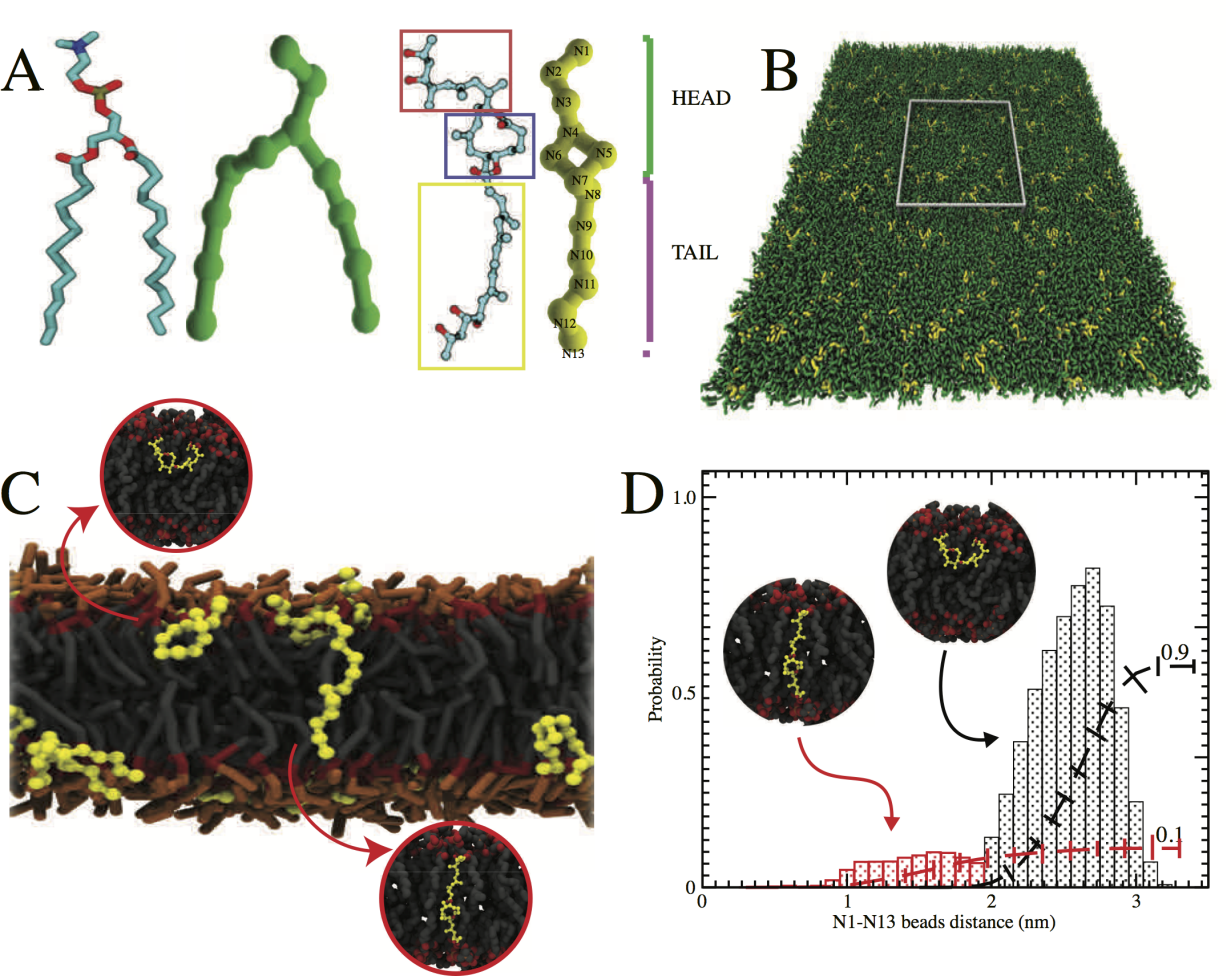
Mycolactone in model lipid membrane. **A)** All-atom and coarse-grained representations of diC16-PC (cyan and green) and mycolactone (cyan and yellow). The atomistic representation is characterized by an 8-undecenolide region (blue box), the C12-C20 northern fragment (red box), and the pentanoic acid ester southern fragment (yellow box). The CG representation can be described in terms of the head and tail regions. Different bead types (N1-N13) capture the general topology of the CG resolution, according to the definition of MARTINI force field (see Methods). **B)** The CG set-up of pure diC16-PC bilayer system in combination with 5% mycolactone. Notice that the simulation box is enclosed by the gray square. **C)** Cross section snapshot of the equilibrated membrane simulation. For clarity, diC16-PC lipid has been depicted by its head group (orange), the glycerol moiety (red) and the aliphatic tails (dark gray). **D)** Histograms correspond to probability distribution of two common configurations of mycolactone in lipid bilayer; either at the surface (black) or spanning the bilayer (red). The distributions are calculated as function of the distance between CG beads N1 and N13 for the bilayer system with 5% mycolactone as shown in the set-up of panel B. The dashed lines show the accumulation of population of these two configurations. Insets in both C and D (enclosed in circles) show representative configurations from all atom MD simulations.

Multinuclear NMR experiments, combined with chemical synthesis have provided the chemical structure of mycolactone, which is a macrolide [4,25–27]. It consists of an 8-undecenolide (C1-C11 fragment) substituted at C11 by a nine-carbon atom chain (C12-C20, the ‘northern’ fragment) and at C5 by a pentanoic acid ester (C1'-C16', the ‘southern’ fragment) as shown in **Fig 1A**. Currently, there are hundreds of synthesized derivatives that retain some of the bioactivity of the native compound and that have helped to shed insight on certain aspects of the structure-activity relationship of the toxin. For instance, it has been shown that the southern fragment of mycolactone is a strong determinant of the molecule’s cytotoxicity [28].

In spite of these advances, much remains unknown about mycolactone’s cytotoxic mechanism of action. Mycolactone has been shown to passively permeate through host cell membranes [29], and to be delivered from bacterial to host cells via outer membrane vesicles [30], though the mechanism of vesicle-host cell exchange is unknown. Furthermore, mycolactone’s solubility in aqueous media is poor, yet it is known to bind soluble protein targets [21]. Most importantly, mycolactone evades the host immune response, and it has not yet been possible to elicit antibodies against it with traditional immunization approaches [31], suggesting its availability in circulation is poor. All of these facets beg the questions: how is mycolactone distributed in the host and what role does its likely association with lipids play in its distribution and pathogenicity?

Mycolactone inhibits both the co- and post-translational pathways for protein translocation across the endoplasmic reticulum (ER) [22,23]. This action has been associated with the inhibition of activation-induced production of cytokines leading to T-cell immunosuppression. It exerts immune-suppressive effects by impairing the capacity of T cells to produce cytokines [32] and by negatively affecting lymphocyte homing through the downregulation of L-selectin [17]. A recent study found that mycolactone led to a specific blockade of translocation of nascent proteins across the ER membrane, though it found no disruption of organelle membrane structure [22]. Further analysis showed that mycolactone inhibits the Sec61 translocon in many different ways [23]. These studies lead to the question of whether such an effect could be exerted through interaction of mycolactone with the ER membrane and/or ER membrane-bound channels involved in protein translocation. To address this question, we need to know how mycolactone interacts with the ER membrane and how it could affect the conformations of transmembrane channels.

It has also been reported that mycolactone interferes with the immune response pathways associated with T-cell activation. The T-cell signaling cascade is triggered by the phosphorylation of ITAM motifs in the T-cell receptor by Lck protein kinase [33]. Then the phosphorylated ITAM recruits ZAP-70, which leads to phosphorylation of downstream signaling molecules such as LAT. The dysregulation of T-cell activation pathway by mycolactone was specific to Lck kinase [8]. Specifically, mycolactone exposure led to hyperphosphorylation of Lck. Surprisingly, this effect was mediated without the direct interaction of mycolactone with Lck. It was proposed that mycolactone-induced hyperpolarization is mediated by changing the partition of Lck such that the concentration of Lck is enhanced in a lipid-raft like domain after 30 mins of exposure [8]. Given the lipid-like structure of mycolactone, is it possible that mycolactone affects the microdomains (eg, lipid raft like domains) in cellular membranes and thereby alters the localization of Lck?

For disease management, rapid point-of-care diagnostics are critical, and mycolactone may provide an avenue to diagnose BU. Interestingly, the development of antibodies against mycolactone by traditional immunization approaches has been challenging [31], likely because exotoxins have cytotoxic effects on immune cells. More recently, a B cell hybridoma technology has been used to develop immune-sera and monoclonal antibodies against mycolactone [34]. When such antibodies are pre-mixed with synthetic mycolactone in solution, they are indeed effective in protecting cells from toxicity. However, their protective ability within a host environment has yet to be demonstrated. This brings up the question of whether the toxin is buried in host lipid molecules/assemblies, hiding its antigenic domain during infection.

The consensus among the above experimental studies has been a lack of details about the association of mycolactone to the cellular membrane, and this deficit has hampered efforts to deduce the mechanisms related to its cytotoxicity, pathogenicity and immunosuppression. Both diagnostic and therapeutic development efforts are also facing technical roadblocks because of the lack of knowledge about how the toxin is distributed and trafficked in the host environment. In this context, and given the toxin’s amphiphilic character, it is critical to address how it interacts with lipids and lipophilic carriers. With the recent advancement in high performance computing and efficient algorithms, numerical simulation approaches such as molecular dynamics (MD) simulations are able to fill the gaps in our understanding of molecular mechanisms in spatial and temporal regions which are often inaccessible to experimental techniques.

In this study, we used coarse-grained (CG) molecular dynamics simulations to study the effects of mycolactone in biomembranes. Our results suggest that mycolactone prefers the membrane over the aqueous environment, localizing predominantly at the glycerol-lipid tail interface just under the membrane surface. We also observe that mycolactone alters several dynamical, physical, and mechanical properties of lipid membranes. Finally, mycolactone is able to decrease the line tension between the ordered and disordered lipid domains, and therefore to potentially interfere with the nano-scale ordering of biological membranes [35]. These findings have substantial implications for the toxin’s distribution in the host environment and mechanisms of pathogenicity.

## Results

### Preferential localization of mycolactone in pure lipid bilayer

First we considered the preferential partitioning of the exotoxin mycolactone between the organic (octanol) and water phases. It is worth pointing out that proper assignment of the partition coefficient is critical for the accurate representation of the molecule at the CG level, where the right balance is required in order the keep consistency with the different sets of parameters [36]. We computed the water-octanol partition coefficient (logP_ow_) of native mycolactone using all-atom molecular dynamics simulations (see Methods and **Panel A in S2 Fig**). We found that mycolactone has a stronger preference for the organic phase over the aqueous phase. We obtained a logP_ow_ of 9.0 ± 0.2 and 11.6 ± 0.2, using Thermodynamic Integration (TI) and Bennett’s acceptance ratio (BAR) approaches, respectively. Similar evaluation of logP_ow_ was carried out with the CG topology using the MARTINI force field [36]. The result from the CG representation provided a logP_ow_ of 8.8 ± 0.1 and 9.0 ± 0.2 using TI and BAR approaches, respectively. The values obtained at coarse-grained representation are in excellent agreement with their atomistic counterparts (**Panel B in S2 Fig, and S3 Fig**). The consistency in logP_OW_ between all-atom and CG MD simulations allowed us to use the CG parameters for rapid exploration of the effects of mycolactone in membrane lipid models, at time scales and system sizes not easily attainable with fully atomistic simulations.

Next, we study the preferential localization of a single mycolactone molecule in a fully hydrated diC16-PC bilayer by considering two independent all-atom MD simulations (1 μs each). A single mycolactone was initially placed in the center of the two membrane leaflets (corresponding to 72 lipids per monolayer) and its localization was tracked. In one case, mycolactone moved to the lipid-water interface where the polar groups could interact with the glycerol and lipid head groups. This conformation was stable and remained for the duration of the simulation. In the other case, the toxin aligned with the aliphatic tails of the lipids near the middle of lipid bilayer. This configuration was seen only once. Although the atomistic simulations are able to independently capture two different spatial configurations during the MD runs, obtaining an overall conformational probability of mycolactone with such short AA simulations is challenging. Therefore, we resort to CG simulations to quantify overall probability distributions of potential mycolactone configurations in model lipid bilayers.

Accordingly, we focus on the study of the preferential localization of the toxin in a fully solvated diC16-PC lipid bilayer using the CG representation. We set up this simulation using the MARTINI force field, which applies a mapping of four heavy atoms to one CG bead (interaction site). The CG representations of mycolactone and diC16-PC are shown in **Fig 1A**. The initial setup of the system is also shown, with the simulation box represented by a gray square in **Fig 1B** containing 66 mycolactones (5% total lipid composition). **Fig 1C** depicts an equilibrated configuration from a 2 μs CG simulation of the mycolactone-DPPC system. We observe that mycolactone preferably adopts two configurations when embedded in the bilayer. All mycolactone molecules were placed initially in an aligned conformation with the lipid tails of the membrane. Approximately 10% of the mycolactone molecules remained aligned with the aliphatic tails of the lipid membranes (**Fig 1D**, red histogram), which is consistent with a configuration from the atomistic simulations (small inset). This configuration allows the extended tails of the northern and southern fragments of the mycolactone to closely interact with the glycerol moieties connecting the head group of the diC16-PC lipids. Most of the mycolactones, however, were found in the water-membrane interface, with both tails interacting with the glycerol moieties of the lipid (**Fig 1D**, black histogram). After the initial 0.5 μs, we didn’t observe any further conversion to this state at the water-membrane interface. Considering the better sampling and faster diffusion of the MARTINI based CG representation, the CG simulations can be used to estimate the relative free energy between the two configurations:

$$\frac{P_{\left(inter\right)}}{P_{\left(alig\right)}}=e^{-(\frac{\Delta G}{kT})}$$

where *P*_*(alig)*_ denotes the population in the aligned state and *P*_*(inter)*_ denotes the population of mycolactones close to the interface. The difference in free energy (-5.7 kJ mol^-1^) suggests that configuration at interface is energetically more favorable. Even though CG simulations are able to populate both configurations, the exchanges between these configurations are still limited by lack of sampling. The potential of mean force calculations as described below provide a more quantitative measure of preference between these two configurations.

**Fig 2:**
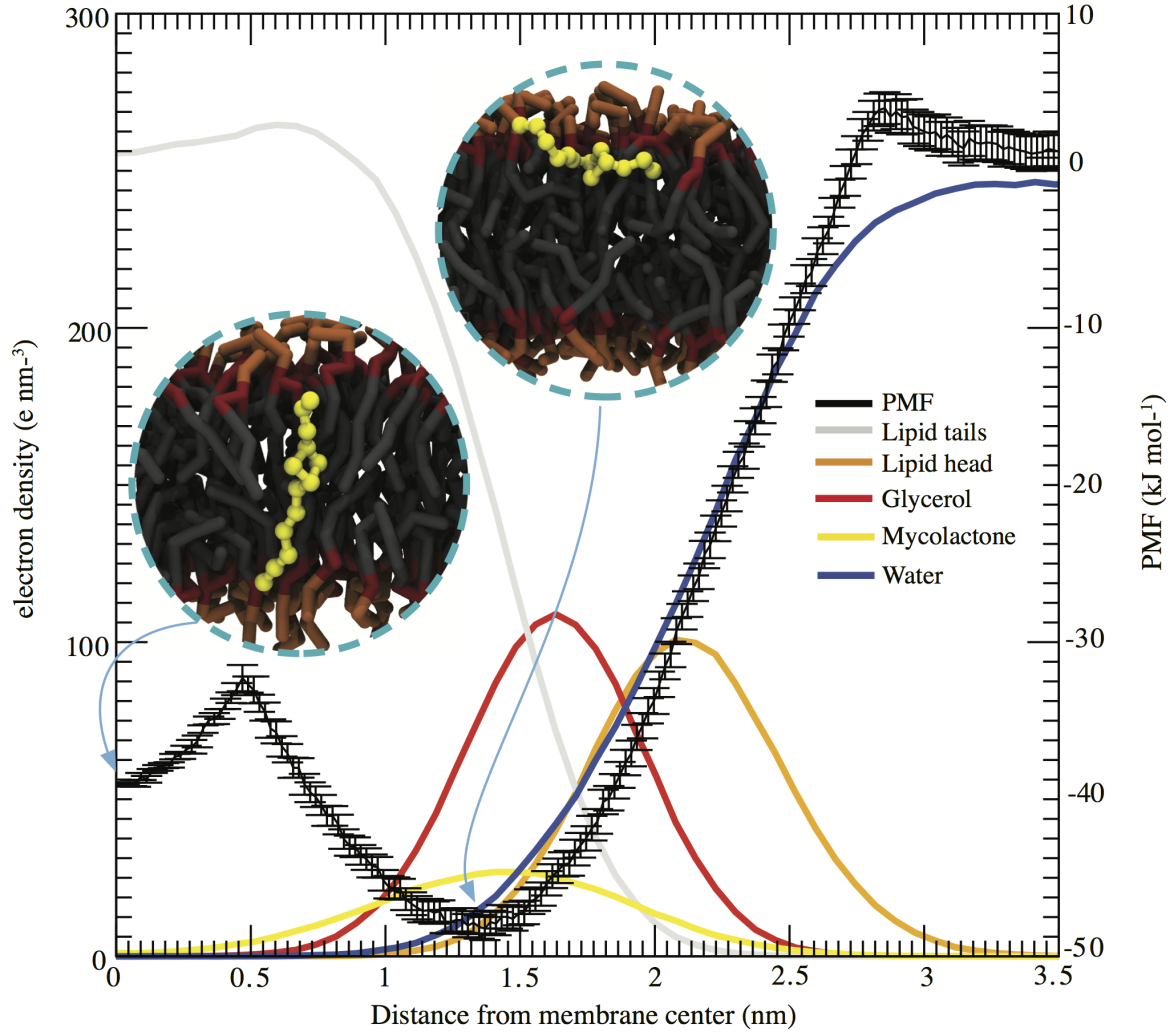
Average electron density profiles and potential of mean force (PMF) for membrane translocation. The electron density (left axis) peak representing mycolactone (yellow line) is localized within the region of the lipid glycerol moiety (red line). Electron-density peaks corresponding to the heads and tails of the diC16-PC lipid are colored as orange and gray respectively. Water density is showed as a blue line. The PMF (right axis) suggests that the preferential localization of mycolactone is in this region (with a value of ~ -50 kJ mol^-1^). A second minimum appears close to the bilayer center (-40 kJ mol^-1^). Overall, the extraction of mycolactone to the bulk water requires 50 kJ mol^-1^. Enclosed insets show representative configurations for each minima, which agree with those observed in unbiased simulations.

To gain more insight into the thermodynamic behavior of mycolactone in the lipid membrane, we calculated the potential of mean force (PMF) for translocation of the toxin through the membrane. The reaction coordinate for translocation followed the axis parallel to membrane normal, tracking the distance between the center of mass of mycolactone and the center of mass of the membrane. Results for one leaflet are summarized in **Fig 2**. As shown, mycolactone is preferentially localized within the boundaries of the glycerol moieties, captured by the minimum at 1.5 nm from the bilayer center. The inset shows the mycolactone conformation that populates this minimum in PMF near the membrane-water interface. This conformation is similar to the one obtained using unbiased simulations. A second minimum is found near the center of the bilayer. This configuration is featured by the membrane aligned structure (**inset**) and stabilized by an energy barrier of ~ 10 kJ mol^-1^. The full detachment of the toxin requires ~ 50 kJ mol^-1^ from the most stable configuration. This value is comparable to the energy required for the extraction of a single cholesterol molecule from a lipid bilayer, suggesting that membranes indeed provide a favorable energetic environment for the toxin. In addition, the PMF shows that the configuration at the interface is favored by ~10 kJ mol^-1^, which is ~4.3 kJ mol^-1^ higher than the direct observations from unbiased simulations.

An effective association constant to the membrane can be calculated by integrating the PMF reported above along the reaction coordinate to the limit of association between the membrane and mycolactone. The association of mycolactone with a lipid membrane is best described as an adsorption or partitioning process. However, one can consider this effective association affinity as a measure of the toxin’s strength of association with membranes, compared to that for its cytosolic targets (e.g., WASP). Consistent with previous membrane association analysis, we use a mathematical formalism [37] for an non-specific association constant:

$$K_{\mathrm{ns}}=\frac{{\left[P\right]}_{mem}}{{\left[P\right]}_{aq}}=\frac{(c^{⊖}{)}^{1/3}\int_{mem}exp[-\beta W(z)]dz}{\int \delta (z-z^{*})\exp\left[-\beta W(z)\right]dz}$$

Where *z* corresponds to the reaction coordinate distance between the center of mass of mycolactone and the bilayer during the PMF. Here, *β* = 1/*k*_*B*_*T* and [*P*] is the concentration of the biomolecule either in aqueous (*aq*) solution or in the membrane (*mem*). z* corresponds to a chosen point on the binding pathway when the biomolecule is far away from the membrane-water interface (e.g. bulk water). The standard state concentration *c*^⊖^ of 1/1660 Å^-3^, corresponds to the standard concentration of 1 M in bulk water, and is raised to the 1/3 since only one degree of freedom (z) is integrated over in the PMF. Notice that the integral over the membrane region in the numerator has units of distance, and must be set relative to the *z* dimension of freedom in the bulk standard concentration, leaving the association constant unitless, as it should be [38,39]. Although they cannot be directly related, the computed nonspecific association constant (3.9 x 10^7^) is on par with the experimentally measured specific association constant of mycolactone for N-WASP (5.8 x 10^6^), and thus clearly reflects a strong association of mycolactone with our model membrane.

The most relevant quantity physiologically is the proportion of toxin buried in membranes relative to that bound to cytosolic targets. One can estimate this proportion based on the approximate size of a cell and concentration of WASP. For a model neutrophil, for example, with a radius of 4.15 μm and a 9 μM concentration of WASP, there would be ~238 molecules of mycolactone bound to the membrane for every one bound to WASP (see **S1 Text** for details). Although this is an approximate estimate for a model membrane (pure DPPC lipid bilayer), it strongly suggests coexistence of the toxin in association with lipids and cytosolic targets. The physiological relevance of this coexistence cannot be overlooked.

### Mycolactone affects the dynamical, mechanical and physical properties of membrane

**Fig 3:**
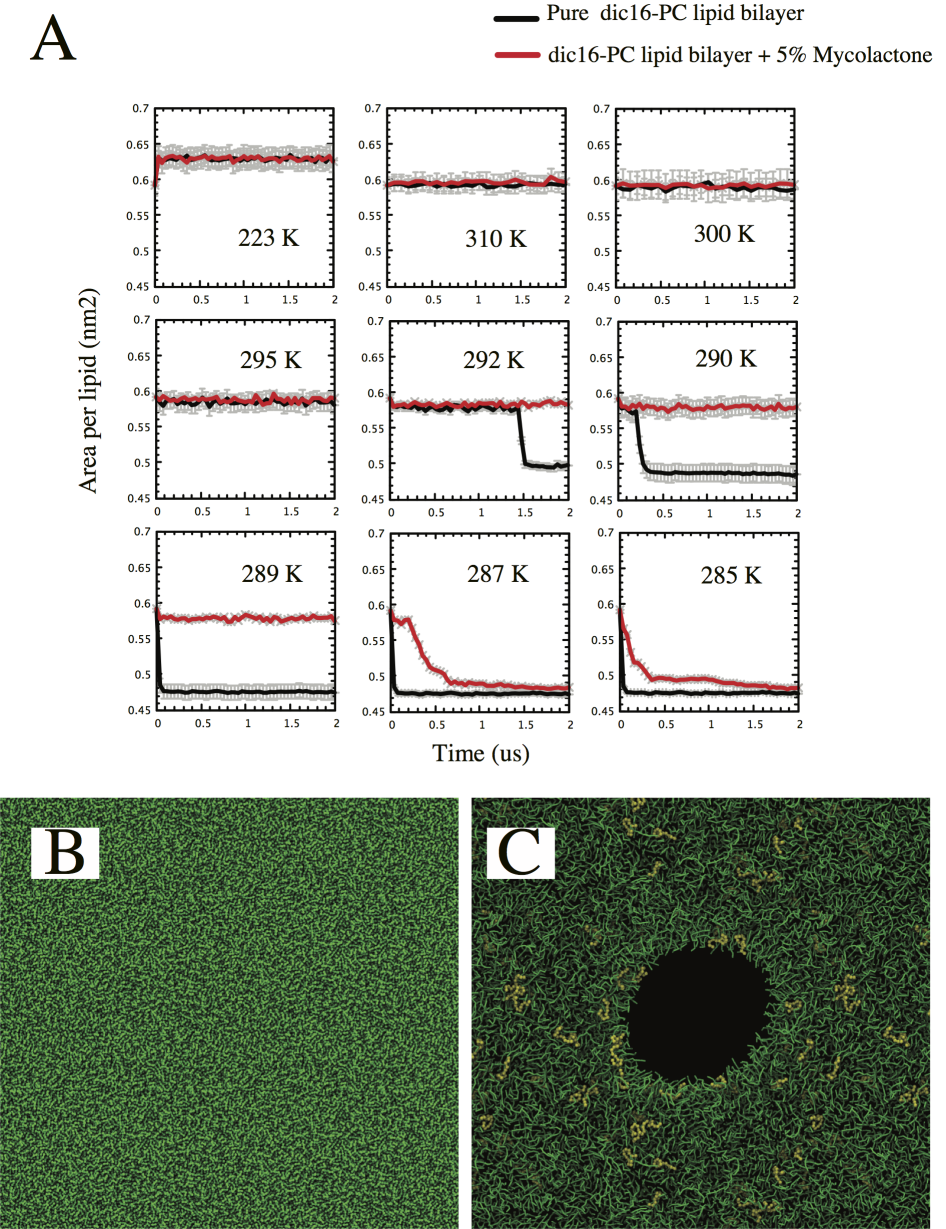
Mycolactone modifies different physical properties of a lipid membrane. **A)** gel-liquid transition temperature for a pure diC16-PC lipid bilayer (black line) and when combined with 5% mycolactone (red line). The transition is given as the change in area per lipid. As previously referenced[40], in the pure diC16-PC membrane, our data shows that gel-liquid transition occurs at ~295 K and with a mean area per lipid of 0.6 nm^2^. However, the addition of mycolactone drops this temperature by ~ 5 K. **B)** Pure diC16-PC lipid bilayer under 60 mN/m surface tension. The membrane thins, but no pore formation is observed. **C)** The addition of 5% mycolactone reduces its resistance to stretching, leading to the formation of a pore with the concomitant rupture of the bilayer at 55 mN/m surface tenstion. Once formed pores were observed to be stabilized at ~ 20 mN/m tension. Pore formation was observed in several independent simulations.

Next, we performed long timescale comparative CG MD simulations of a mycolactone-diC16-PC system and a pure diC16-PC lipid bilayer system. In addition to our PMF calculations, which suggested a preference of mycolactone towards lipid membranes, the toxin’s influence on the structure and/or dynamics of lipid bilayers was also of interest. This can be characterized through comparative simulations with and without the mycolactone. First, 2 μs simulations are used to investigate the effect of mycolactone on the transition melting (Tm) temperature of a pure diC16-PC bilayer. As shown in **Fig 3A**, the presence of mycolactone reduces the gel-liquid Tm of diC16-PC by ~5 K, stabilizing the liquid phase at lower temperatures. When the mycolactone-diC16-PC system is cooled from 323K, the formation of a gel phase is observed at 287K. When mycolactone was not present, the pure diC16-PC system transitioned towards the gel phase at a slightly higher temperature. The liquid phase is observed at 292K, which is in agreement with the reported value in the original model [40]. In the simulations, the formation of gel phase is captured by the drop in the area per lipid which converges to an averaged area per lipid of ~ 0.47 nm^2^ in both systems. Above these transition temperatures, both systems display similar area per lipid (0.64 nm^2^). We also measured the lateral diffusion constants at 323 K and these values are similar for both systems (1.7 x 10^−7^ cm^2^ s^-1^ and 2 x 10^−7^ cm^2^ s^-1^ for the pure and mixed systems, respectively).

Another important feature under consideration was the area compressibility modulus *K*_*A*_ which can inform on membrane deformations due to stretch. We calculated *K*_*A*_ (see Methods) considering a large patch of ~ 6000 diC16-PC lipids with either 5% or 10% mycolactone content. Our results suggest that mycolactone strongly influences the membrane resistance to compression. For pure diC16-PC bilayers, the area compressibility modulus is *K*_*A*_ = 282 ± 60 mN/m. This value compares reasonably well with the experimental value, which was reported to be *K*_*A*_ = 231 ± 20 mN/m [41]. The compressibility modulus is reduced, however, when 5% mycolactone is incorporated in the bilayer (*K*_*A*_ = 208 ± 30 mN/m). Addition of more mycolactone (up to 10%) decreases the *K*_*A*_ even more, to a value close to 99 ± 50 mN/m. Thus, compared to a pure lipid bilayer, lower force can rupture the membrane when mycolactone is present.

Then, we examined whether mycolactone can affect the role of biological membranes as barriers by increasing their tendency to be porous. Similar comparative simulations were used to compute the effect of mycolactone on the critical line tension (see Methods) of the diC16-PC bilayer. Under low tension, mechanically generated pores are prone to close (fill up). Under high tension, however, pores tend to grow larger, eventually causing rupture of the membrane. Typically, highly ordered lipid bilayers have a high edge energy (line tension) compared with more elastic membranes. **Fig 3B** shows the effect of applying ~ 60 mN/m surface tension to a pure diC16-PC bilayer. After 2 μs, the membrane became thinner, but no pore formation was observed. However, the membrane containing 5% mycolactone, was rapidly stretched, followed by the spontaneous formation of a pore (**Fig 3C**). Similar behavior was also observed in 10 independent simulations. However, when the applied surface tension was reduced to 55 mN/m, pore formation was not observed. This suggests a decrease of at least ~8% in the critical line tension. Furthermore, in pure diC16-PC bilayer, pre-formed pores are stabilized at ~ 25 mN/m, however we found that this value decreases to ~20 mN/m when the membrane interacts with mycolactone. A proper regime of different surface tensions needs to be sampled to obtain a more quantitative value of line tension and to profile the timeline of formation and evolution of pores. Even though mycolactone could directly alter the surface tension, that effect is very small at the given concentration. However, in an already thinned membrane, 5% mycolactone affects the surface tension enough to form pores.

### Mycolactone modifies the line tension between lipid domains in a mixed lipid bilayer system

The lateral heterogeneity of biological membranes likely plays an important role in cellular biophysics. For example, the activity of proteins localized within different domains can be influenced by the local membrane properties, and thus by phase properties. In model membranes, ternary mixtures of saturated lipids, unsaturated lipids, and cholesterol are known to segregate into two coexisting fluid lipid domains, liquid-ordered (Lo) and liquid-disordered (Ld) [42]. A line tension exists at the interface between such domains. Linactants are molecules that can modify the equilibrium in the boundaries by modulating the line tension between these domains and shift the preferential segregation of the molecules embedded in such regions.

By comparing simulations of a ternary lipid mixture with and without mycolactone, we evaluated whether mycolactone can act as a linactant. To achieve this, we set up a control system composed of randomly placed diC16-PC, diC18:2-PC and cholesterol (4:3:3 lipid ratio) as described in the Methods section. At the CG level, this system has previously been observed to segregate into liquid ordered and liquid disordered domains on a simulation time scale of 2 μs [43]. In addition, we also set up a mixed ternary system with 5% (of total lipid) made up of mycolactone. Both simulations were run for a total of 10 μs. Considering that the MARTINI model speeds up diffusion by a factor of ~4 [36], processes longer than 10 μs should be captured in these simulations as well. Both systems were started with a random lipid distribution.

**Fig 4:**
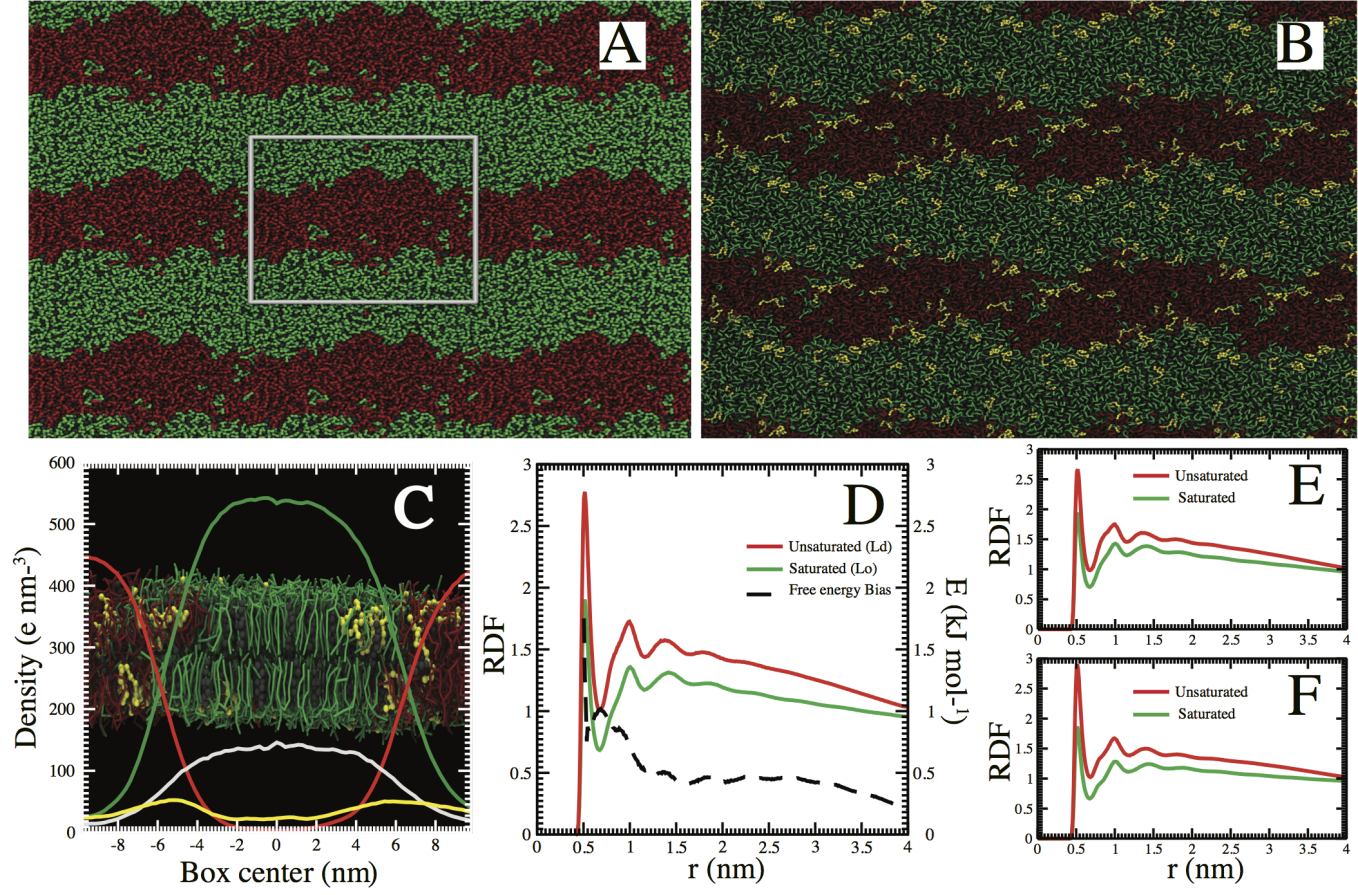
Mycolactone acts as a linactant. **A)** Spontaneous Lo (green lipids)-Ld (red lipids) domain formation in a ternary lipid system (diC16-PC, diC18:2-PC and cholesterol) at 4:3:3 lipid ratio. **B)** The addition of 5% mycolactone, however, decreases the line tension between the domains (see main text). **C)** Electron density profile along the interface of both domains. Clearly the higher peak corresponding to mycolactone (yellow line) is localized in the interface of both domains. Green and red lines highlight the electron-density profiles for the ordered and disordered lipids (diC16-PC and diC18:2-PC respectively). The white line corresponds to the electron-density of cholesterol within the Lo and Ld domains. **D)** Radial distribution function of mycolactone with the center of mass of ordered lipids-saturated tails (green line) and disordered lipids-unsaturated tails (red line) clearly show a slight preference for the disordered region, which is expressed by the free energy difference between the red and green (black dashed line). The radial distributions were also split by considering the head **(E)** and tail **(F)** regions of mycolactone, suggesting that both regions prefer the Ld region.

In the absence of mycolactone, the ternary system undergoes lipid segregation (**Fig 4A**), where cholesterol rich-domains are surrounded by saturated lipid tails. Cholesterol-poor domains are localized in the Ld region containing unsaturated lipid tails. It took about 5 μs for these domains to equilibrate, and no further transition was observed. We quantified the line tension between the two domains (see Methods), considering the last 5 μs of the trajectory. In line with previous calculations [44], the membrane patch considered here converged to a line tension of 16 ± 1.3 pN.

Under similar simulation conditions, the membrane patch containing 5% mycolactone (**Fig 4B**) converged to a value of 7.2 ± 0.5 pN. This decrease of line tension by ~50% is comparable with the effect observed with different linactants [44]. To show the preferential localization of mycolactone in the membrane, we computed the electron-density profile along the interface of both domains in the membrane. As depicted in **Fig 4C**, the slightly higher peak corresponding to mycolactone is localized within the boundaries of the Lo and Ld, suggesting that indeed mycolactone co-localizes within the interface of both domains and may act as a linactant.

We further quantified the lipid interaction preference, computing the lateral radius distribution (RDF) of mycolactone with respect to the center of mass of the saturated and unsaturated lipids, respectively. As shown in **Fig 4D**, mycolactone preferentially interacts with the unsaturated lipids from the Ld domain. The electron density profile shows a preference of ~70% for the interface region compared to bulk Ld and Lo regions. The preferential lateral interaction free energy (ΔG_PLI_) of mycolactone with the saturated versus unsaturated lipids is ~2 kJ mol^-1^. As shown in **Fig 4D**, energetically, the direct interaction with mycolactone is slightly preferred for unsaturated lipids over saturated lipids. Values within the same range have been reported previously for the preferential partitioning of other lipid species [45]. **Fig 4E and 4F** show that similar preference is maintained for the head and tail regions of the molecule. Given the low ΔG_PLI_ (in the range of *kT*), our results suggest that mycolactone is preferentially localized within the interface. Such a mechanism may allow it to decrease the line tension and drop the energy barrier that keeps both domains stable.

## Discussion

The pathogenesis of Buruli ulcers, caused by *Mycobacterium ulcerans*, is clearly tied to mycolactone, a lipid-like exotoxin capable of permeating the host cell membrane, binding to cytosolic and membrane-bound targets, and ultimately inducing cell death. Although much has been learned about mycolactone’s mechanism of toxicity, many questions remain. In this study, we focused on the interactions of mycolactone with models of biomembranes, hoping to shed light on the toxin’s distribution and mechanisms of host cell penetration.

The computed water-octanol partition coefficient indicates that mycolactone prefers to be in an organic solvent rather than an aqueous environment. Furthermore, our free energy calculation reveals that the exotoxin is preferentially buried in a pure lipid bilayer, in agreement with the partitioning data. In addition, both the unbiased and enhanced sampling simulations suggest that once in the bilayer, mycolactone preferentially localizes around the lipid glycerol groups close to the lipid-water interface, but that it can also span the membrane, reaching out to interact with the polar groups on either leaflet. This suggests a plausible mechanism for its translocation from the extracellular to the cytoplasmic side of the plasma membrane in which is localizes on one leaflet’s lipid-water interface, then spans, then flips to localize on the other leaflet’s lipid-water interface. There have been no experimental measurements of mycolactone in lipid versus solvent mediums to directly compare to our findings. Although it was reported that a fluorescent derivative of mycolactone accumulates in the cytosol of murine cells [29], the reported images suggest localization within the ER membrane. Our simulation results are also indirectly supported by the composition of the native toxin extracted from infected patients. Those studies report that the toxin is heavily bound to the lipid extracts of the cell, suggesting that mycolactone is, in the absence of higher binding affinity host proteins, localized within the non-polar phase of the cell (e.g., membranes) [18,46].

Mycolactone clearly needs to translocate across cellular membranes to reach its cytosolic and ER targets [18,47]. Given that our study shows that the exotoxin has a preference for membrane relative to aqueous environments, it is suggested that membranes and other lipid carriers facilitate its translocation throughout the host environment. Some of these carriers include cytosolic proteins, such as WASP and N-WASP. The mechanism for exchange from membrane to host cytosolic carriers could be further facilitated by co-localization, since targets like WASP and N-WASP are recruited to the surface of cell membranes by membrane-bound or membrane-associated proteins prior to activation by effector molecules (e.g., CDC42). This is supported by the computed association constant between mycolactone and a model lipid membrane being on the same order or slightly stronger than the measured association constant with N-WASP [21]. Importantly, the relative affinity of mycolactone for the membrane will depend on the membrane composition. It is expected that the exotoxin prefers intracellular membranes, such as the endoplasmic reticulum, but this will have to be verified in future studies. Regardless, a relatively strong affinity to the plasma membrane would help to increase the local concentration of mycolactone on the host membrane, directing its flow toward intra-cellular targets (e.g., WASP, the endoplasmic reticulum, and Sec61). Thus, our findings support a passive diffusion mechanism of cellular access for mycolactone, as reported [29], but further suggest that membrane localization could be playing a direct role in its uptake into intracellular membranes and handoff to cytosolic targets.

It is interesting that our CG MD simulations suggest a mycolactone-dependent membrane disruptive effect, albeit at reasonably high toxin concentrations. In fact, all of our calculations demonstrate that the addition of the exotoxin perturbs both structural and dynamic properties of the lipid bilayers. The addition of mycolactone lowers the crystalline-gel Tm by ~5 K in a diC16-PC bilayer, which is associated with the preservation of the fluid phase at lower temperatures. We further associate this reduction in Tm to the disruption of lipid interactions, especially within the glycerol moiety region. Similar effects have been attributed to disaccharides [48], although the critical concentration is several orders of magnitude higher.

From the biological perspective, the observation that mycolactone affects the elastic properties of the model diC16-PC lipid membrane is of relevance. Our calculations suggest a reduction of ~30% and 70% of the compressibility modulus with the addition of 5% and ~10% mycolactone, respectively. Furthermore, mycolactone reduces line tension of membranes, including the critical line tension that reports on the porosity of membranes. The magnitude of changes in membrane properties caused by mycolactone could, in principle, influence the activity of membrane-embedded proteins [49]. Previous studies have addressed how changes in membrane elastic properties can affect glucose transporters [50], the stability of voltage sensor segments [51], mechano-sensitive channels of large conductance [52], and clathrin protein ordering [53]. However, the most likely connection between membrane association and Sec61 inhibition is that trafficking to the endoplasmic reticulum could facilitate mycolactone binding to the Sec61 translocon.

In the context of our model biological membrane with ordered and disordered domains, mycolactone potentially behaves as a linactant. Addition of this toxin results in a marked reduction in the lateral line tension between lipid domains in a ternary lipid mixture. Moreover, mycolactone preferentially localizes at the interface between liquid-ordered (Lo) and liquid-disordered (Ld) domains. The mechanism of disruption seems to correlate with an individual destabilization, with no aggregates or mesoscale structures formed [54] at low concentrations. Interestingly, the toxin shows a slight preference to interact with the unsaturated tails of lipids from the Ld domain. However, the toxin does not accumulate in the bulk of the Ld domain, but rather at the interface, as seen in the electron density profile. The preferential localization of mycolactone at the interface between Ld and Lo domains allows mycolactone to decrease the line tension. This linactant activity of mycolactone could impact the intracellular signaling pathways that are coupled to T-cell receptor (TCR) activation. It is possible that mycolactone behaves as a linactant where it can enhance the mixing of disordered and ordered domains, thus promoting the transfer of Lck into a lipid-raft like ordered domain.

Finally, our study provides the first glimpse of how mycolactone interacts with membrane and alters membrane stability and dynamics. It is unclear how the properties described above (i.e., a preferential localization in the lipid phase, particularly at the interface of ordered and disordered lipid domains, and altered biophysical properties of lipid bilayers) relate to the cytotoxic nature of mycolactone [19]. Clearly, they will influence the processes by which mycolactone penetrates the host cell and how it is trafficked, both intra- and extra-cellularly. It is also likely that mycolactone will not be presented during infection as a monomeric, water-soluble molecule; rather it will be bound to and carried by host molecules with hydrophobic domains (e.g., WASP, N-WASP) and/or lipid assemblies (e.g., bacterial vesicles, host cell membranes, and likely other assemblies such as high density lipoproteins). Also, localization at the ordered-disordered interface could assist in the exchange of mycolactone between bacterial outer membrane vesicles and host membranes. Further modeling and biophysical experimental studies are required to clarify these aspects of mycolactone cytotoxicity.

We believe that molecular understanding that we gained on how mycolactone interacts with lipids could help the rational development of diagnostics and adjunctive therapies that target mycolactone as it appears during infection. In fact the development of antibodies that target mycolactone derivatives is well underway [34], but their efficacy in toxin neutralization hinges on mixing the antibodies and mycolactone prior to cell exposure. This again suggests that in a cellular environment, mycolactone is hidden from water-soluble antibodies. Additionally, understanding mycolactone distribution and trafficking may help answer some of the remaining questions about how the toxin kills cells, evades immune responses, and plays a role in angiotensin pathways. Recently, it was shown that mycolactone exerts local analgesia by binding to angiotensin II type 2 receptors and leads to potassium-dependent hyperpolarization of neurons [24]. These findings have led to the exploration of using mycolactone and its derivatives therapeutically to suppress pain and inflammatory responses [55]. Therefore, understanding the nature by which mycolactone interacts with lipids may help in realizing the potential of mycolactone as a therapeutic agent.

In summary, understanding the details of the mycolactone interaction with lipids, its influence on membrane dynamics and stability, and its thermochemical behavior in lipid and aqueous environments will be tremendously useful in understanding the variety of ways in which this toxin induces host disease, in the development of diagnostic tools for Buruli ulcer. Moreover, mycolactone has proven to be a helpful test bed for understanding host-pathogen interactions involving amphiphilic molecules. We used coarse-grained molecular dynamics simulations to explore the molecular behavior of mycolactone in lipid membranes. From simulations of mycolactone in aqueous, pure lipid, and mixed lipid bilayer systems, we find that mycolactone prefers the lipid to the aqueous environment, and that it could potentially perturb the thermochemistry of biological membranes in terms of transition temperature, compressibility, and line tension. Interestingly, mycolactone acts as a linactant, i.e., it localizes at the boundary between different fluid lipid domains in membranes and promotes inter-mixing of domains. It should be kept in mind that in these simulations, we haven’t considered the diversity of components present in a typical biological membrane, but rather very simple models of lipid membranes. Regardless, it is striking to observe disruptive effects induced by this toxin on a lipid bilayer system. We speculate that such effects could easily translate to a broad number of biochemical signatures typical of the pathogenesis of the Buruli ulcer disease [8,22,24]. In this context, we suggest further studies, both experimental and theoretical, that can capture the effects of mycolactone in more complex systems in which the relevant biological components are present.

Note added in proof: Subsequent to the acceptance of this paper we became aware of a recent independent publication that provides experimental confirmation of our prediction that mycolactone would decrease the line tension and thereby disrupt lipid domain formation [56].

## Methods

### Mycolactone model

The structural coordinates of mycolactone were downloaded from the PubChem database (CID 5282079). Mycolactone is structurally inhomogeneous, with variants showing a common core macrocycle and differences in the southern acyl chain. The best studied is mycolactone A/B which is prevalent in Africa, Malaysia, and Japan. The A and B isomers of mycolactone differ by 180^0^ rotations about the C4’-C5’ double bond and C5’-C6’ **single bond** in the southern chain; they are in dynamic equilibrium with a 60/40 ratio of mycolactone A to B [57]. Here, we focus our studies on mycolactone B since that conforms to the structural form of mycolactone deposited in the PubChem database. We refer to mycolactone B simply as mycolactone in the manuscript (**Fig 1A**).

### Molecular dynamics simulations set-up

#### Atomistic system

To properly assess the behavior of mycolactone between the organic and aqueous phases, we calculated the water-octanol partition coefficient. For that purpose, the mycolactone molecule was placed in a 3.5 nm cubic box and solvated with either water molecules or octanol. Water molecules were represented using the TIP3P water model. Mycolactone and octanol molecules were represented at all-atom level using the General AMBER force field (GAFF) [58]. Partial charges of atoms were optimized using the restrained electrostatic potential (RESP) approach [47], in line with the AMBER force field parameterization. The final topology parameters are provided in the supporting material (**S1 Table**).

To study the preferential localization of mycolactone in the membrane environment, small atomistic lipid patches (6.5nm x 6.5nm) were used. To build the system, a single mycolactone was manually embedded in a pre-equilibrated Dipalmitoylphosphatidylcholine (diC16-PC) lipid membrane patch. Overlapping diC16-PC lipids and water molecules were removed in order to avoid unwanted higher forces. The system was posteriorly equilibrated above the lipid phase transition temperature (323 K) for 20 ns. Lipids were represented using the updated AMBER lipid force field [59].

All-atom (AA) MD simulations were performed using the GROMACS 4.5 molecular dynamics package [60]. Simulations were performed using a 2 fs time step. The LINCS algorithm was applied to constrain all bond lengths with a relative geometric tolerance of 10^−4^. Non-bonded interactions were handled using a twin-range cutoff scheme. Within a short-range cutoff of 0.9 nm, the interactions were evaluated every time step based on a pair list updated every five time steps. The intermediate-range interactions (up to a long-range cutoff radius of 1.4 nm) were evaluated simultaneously with each pair list update and were assumed constant in between. A PME approach was used to account for electrostatic interactions with a grid spacing set to 0.15 nm. Constant temperature was maintained by weak coupling of the solvent and solute separately to a velocity-rescaling scheme [61] with a relaxation time of 1.0 ps. The Berendsen algorithm was used to couple the system pressure at 1.0 bar through an isotropic approach with relaxation time of 1.0 ps. Membrane systems were quenched to 323 K and coupled using a semi-isotropic pressure approach within the XY plane. Trajectories were run for 1 μs and stored every 0.02 μs for posterior analysis.

#### Coarse-grained system

We use the MARTINI force field [36] for representing the CG systems. MARTINI is an empirically parameterized CG force field that enables the simulation of many bio-molecular systems by considering a general parameterization protocol for diverse organic molecules. As stated in the MARTINI approach, new molecules can be parameterized based on AA MD simulations and then validated based on partition coefficients between different organic environments. Thus, for our system, the CG parameters for mycolactone were calculated from the AA MD simulations mentioned above. A two-step approach was carried out. Firstly, the equilibrated atomistic trajectory of a single mycolactone in water (0.5 μs) was converted to a pseudo-CG trajectory using the center of mass of the appropriate fine-grained particles using a 1–4 mapping scheme [62].

$$r_{i}^{CG}=\frac{\sum_{j=1}^{p}r_{j}m_{j}}{\sum_{j=1}^{p}m_{j}}$$

The vector **r**_i_^CG^ describes the position of the pseudo-CG bead, *p* is the number of atoms mapped to a given coarse bead, *m*_j_ is the mass of the atom *j*, and **r**_*j*_ is its coordinates. From the atomistic simulation the target distribution functions (e.g. bonds, angles, dihedrals) were obtained and iteratively introduced into the set of CG parameters. Secondly, for a proper assigning of the MARTINI beads, we iteratively calculated the water-octanol partition coefficient (see next section). The optimization of CG parameterization was reached when the value was in agreement with the calculated from the AA MD simulations. The final topology parameters for both AA and CG models are provided in the supporting material (**S1 Table**).

For CG MD simulations, lipid membranes were represented using the MARTINI CG force field [36] as described above. A full description of the different membrane compositions is provided in **Table 1**. Briefly, pure diC16-PC bilayers where built using the ‘*insane’* script (a molecular and geometrical construction algorithm) [63], consisting of ~1300 lipids. The membranes were fully solvated at a proportion of 35 CG water molecules per lipid, and 5% mycolactone (molar/molar) was added to the membrane. Bigger CG membrane patches (~6000 lipids) were built in order to capture the undulation modes in the membrane. In this case we tested 5% and 10% mycolactone concentrations. For CG MD simulations of DPPC at temperatures below its Tm (290K), anti-freeze particles (0.1 mol particles) were added. The effect of mycolactone on the domain line tension was studied by building a quaternary lipid system consisting diC16-PC, diC18:2-PC, cholesterol (CHOL), and mycolactone. Lipids diC16-PC and diC18:2-PC and cholesterol were distributed according the ratio of 4:3:3, respectively. Lipids were laterally and randomly placed in a triclinic box of edges 25x35 nm. A summary of all the systems and simulations performed in this work is presented in **Table 1**.

**Table 1:** Summary of the CG MD simulations carried out in this study.

System	Composition	Temperature (K)	Pressure (bar)	Simulation time* (μs)	Number of runs
**Transition temperature**					
diC16-PC	1352 lipids, 11867 CG water	285, 287, 289, 290, 292, 295, 300, 323	1	2	1 per temperature
diC16-PC and Mycolactone	1284 lipids, 66 Mycolactone, 11593 CG water	285, 287, 289, 290, 292, 295, 300, 323	1	2	1 per temperature
**Compression modulus**					
diC16-PC	6728 lipids, 74865 CG water	323	1	2	2
diC16-PC and Mycolactone 5%	6390 lipids, 336 Mycolactone, 73676 CG water	323	1	2	2
diC16-PC and Mycolactone 10%	6054 lipids, 672 Mycolactone, 72532 CG water	323	1	2	2
**Doman line tension**					
diC16-PC, diC18:2-PC, CHOL	828 diC16-PC, 540 diC18:2-PC, 432 CHOL, 15589 CG water	295	1	10	1
diC16-PC, diC18:2-PC, CHOL and Mycolactone	788 diC16-PC, 514 diC18:2-PC, 410 CHOL, 84 Mycolactone, 15330 CG water	295	1	10	1
**Edge energy and pore stability**					
diC16-PC	1352 lipids, 11867 CG water	300	-24 to -50	2	2
diC16-PC and Mycolactone	1284 lipids, 66 Mycolactone, 11593 CG water	300	-24 to -50	2	2
**Free energy Mycolactone-membrane translocation**					
diC16-PC	256 lipids, 1 Mycolactone, 5000 CG water	323	1	10	40

* Note that the actual simulation time is reported (not the effective simulation time including the 4-fold speed up often reported for MARTINI CG simulations).

The CG lipid membranes were equilibrated using a general MARTINI protocol. In brief, the non-bonded interactions are cutoff at a distance rcut of 1.2 nm. To reduce the generation of unwanted noise, the standard shift function of GROMACS is used in which both the energy and force smoothly vanish at the cutoff distance. The global dielectric constant was adjusted to ε = 15. Non-bonded interactions are cut off at a distance of r_cut_ = 1.2 nm. In addition, the LJ potential is shifted from r_shift_ = 0.9 nm to r_cut_, and the electrostatic potential is shifted from r_shift_ = 0.0 nm to r_cut_. The time step used to integrate the equations of motion is 20 fs. Constant temperature is maintained by weak coupling of the solvent and membranes separately to a Berendsen heat bath with a relaxation time of 1.0 ps. During equilibration, bilayers were coupled to 1.0 bar using a semi-isotropic barostat with a relaxation time of 1.0 ps. After equilibration, membranes were adjusted using the Parrinello barostat set at 1.0 bar. Temperature was set to 323K, except for the calculation of transition temperature (Tm), in which membranes were quenched from 285K to 295K. Stable membrane domains were equilibrated at 295K, as has been previously shown [43].

### Partition coefficient (logP) and potential of mean force (PMF)

The octanol-water partition coefficient (logPow) for mycolactone was calculated for both AA and CG systems. Similar calculated logPow will ensure a consistent representation of the model in both representations. Given the appropriate free energy of solvation, the computation of the partition coefficient is straightforward. The difference between the solvation free energy in aqueous (ΔG_W_) and octanol (ΔG_O_) phase is the partitioning free energy (ΔΔG_OW_);

$$\Delta \Delta G_{OW}=-2.3RTlogP_{OW}$$

where *R* and *T* correspond to the universal gas constant and the temperature of the system, respectively. ΔG_W_ and ΔG_O_ were calculated directly by uncoupling the non-bonded interactions of the solute with the respective solvent using the thermodynamic integration approach:

$$∆F_{BA}=F_{B}-F_{A}=\int_{\lambda A}^{\lambda B}d\lambda \langle \frac{\partial Uuv(\lambda )}{\partial \lambda }\rangle \lambda$$

Here λ is a coupling parameter that regulates the strength of the interaction of *F*_*B*_ (fully uncoupled) and *F*_*A*_ (fully coupled). U_*uv*_(λ) denotes the potential energy function describing the total solute-solvent interaction. The average <…> is taken over the MD trajectory. Calculations were performed at 25 independent λ points. For each individual λ, simulations were run for 50 ns (AA) or 100 ns (CG) respectively.

The obtained water-octanol partition coefficients were compared with values obtained using the Bennett’s acceptance ratio[64]. The results are highlighted in **S3 Fig** for both octanol and water solvation free energies using the CG and AA simulations. In general, we observe that both approaches provide similar logP values, although the latter slightly increases the preference for the organic phase at AA resolution.

The membrane translocation potential of mean force (PMF) by mycolactone was calculated using the umbrella sampling approach on the CG system. The simulation was composed of 40 independent windows spaced by 1 Å. A restraining potential of 1000 kJ mol^-1^ nm^-2^ was applied to the center of mass of the entire mycolactone with respect of the center of mass of the lipid bilayer and along the normal (z) coordinate. For each window, 10 μs long simulations were performed. We should state, however, that MARTINI based simulations lead to an effective speed up of a factor of ~4 [36], effectively giving a 40 μs time per window. PMFs were reconstructed using the weighted histogram [65] approach and convergence was assessed using the bootstrap method. Also, we assessed the convergence of the calculations using block averaging where the trajectories for each window were divided into independent blocks of 1 μs each. The different PMFs were calculated for each point and averaged. These results are presented in **S1 Fig** and in good agreement with the bootstrap method.

### Line tension (edge energy) and critical line tension

The line tension, or edge energy, of the lipid membrane can be obtained from the critical line tension at which pores can be stabilized inside a membrane. According to a theoretical model [66]:

$$E(r)=2\pi \lambda -\pi r^{2}\gamma$$

where E(*r*) = energy of a pore of radius *r* inside a membrane, λ is the line tension that opposes the pore formation, and γ is the surface tension that reduces the energetic barrier for pore formation. At low tension, pores are unstable, however, at a critical line tension γ*, the edge energy can be overcome. Thus, pores can be stabilized at r = γ*/λ.

The line tension at the interface of two different lipid domains, σ, can be obtained from the bulk pressures measured during the simulation. For an interface along the X-dimension, σ can be obtained through the following expression [44]:

$$\sigma =\frac{1}{2}\langle L_{Y}L_{Z}(P_{YY}-P_{XX})\rangle$$

where <…> denotes an ensemble average, L_Y_ and L_Z_ are the box dimensions along the Y and Z edges respectively, and P_YY_ and P_XX_ are the pressure tensors perpendicular and parallel to the interface. The factor ½ accounts for the fact that there are 2 interfaces in the simulation system.

### Compressibility modulus

The area compressibility modulus, *K*_*A*_, defined within one plane can be calculated from the fluctuations in the membrane area per lipid [67]:

$$K_{A}=\frac{kT<A_{0}>}{N<(A-A_{0}{)}^{2}>}$$

where *N* denotes the number of lipids per monolayer, *A*_0_ is the averaged area per lipid and *A* the fluctuation of the area per lipid from the simulation trajectory. *kT* is 2.49 kJ mol^-1^ at 300 K. It has been observed that better agreement with experimental data is found for simulations of large membrane patches, as undulation modes can be captured.

## Supporting information

S1 Table Parameters for the simulation of Mycolactone. (PDF)

S1 Fig Potential of mean force (PMF) of mycolactone translocation from diC16-PC bilayer. In addition to the plot reported in the main manuscript, the error in the calculated PMF was obtained through block averaging. Thus, independent PMFs were obtained from trajectory blocks of 1 μs (dash lines). The total PMF was obtained from total averaging (solid black line).
(PDF)

S2 Fig Thermodynamics integration profiles as function of the integration parameter *λ* for mycolactone in water and in octanol. **A)** Snapshots of mycolactone in either water (left panel) and octanol (right panel) simulation boxes. **B)** Running derivatives for thermodynamic integration of mycolactone atomistic representation (top panels) and coarse-grained representation (bottom panels). Red lines correspond to the integral.
(PDF)

S3 Fig Calculation of the solvation free energies using the Bennett’s acceptance ratio. The λ dependency was computed using the g_bar tool as implemented in GROMACS and logP values (red = AA, black = CG) were obtained using equation referenced in the main manuscript.
(PDF)

S1 Text Membrane association constant and cellular distribution of mycolactone. (PDF)
