# Supplementary material for: Membrane perturbing properties of toxin mycolactone from Mycobacterium ulcerans
Source: PLoS Comput Biol. 2018 Feb 5;14(2):e1005972. doi: 10.1371/journal.pcbi.1005972 (PMC5814095; doi:10.1371/journal.pcbi.1005972)
Supplement: S1 Table — (PDF) [file pcbi.1005972.s001.pdf]

Membrane perturbing properties of toxin mycolactone from *Mycobacterium ulcerans*

Cesar A. López, Cliff Unkefer, Basil Swanson, Jessica M. J. Swanson, S. Gnanakaran

"*Supporting material*"

Table S1. Parameters for the simulation of Mycolactone

Atomistic

```
[ moleculetype ]
;name            nrexcl
 MYCO            3

[ atoms ]
;   nr  type  resi  res  atom  cgnr     charge      mass
     1   os     1   MYCO     O    1    -0.445900     16.00000
     2   os     1   MYCO    O1    2    -0.448900     16.00000
     3   oh     1   MYCO    O2    3    -0.596801     16.00000
     4    o     1   MYCO    O3    4    -0.538001     16.00000
     5   oh     1   MYCO    O4    5    -0.598801     16.00000
     6    o     1   MYCO    O5    6    -0.541001     16.00000
     7   oh     1   MYCO    O6    7    -0.609801     16.00000
     8   oh     1   MYCO    O7    8    -0.593801     16.00000
     9   oh     1   MYCO    O8    9    -0.605801     16.00000
    10   c3     1   MYCO     C   10    -0.071700     12.01000
    11   c3     1   MYCO    C1   11    -0.072700     12.01000
    12   c3     1   MYCO    C2   12     0.159100     12.01000
    13   c3     1   MYCO    C3   13     0.151100     12.01000
    14   c3     1   MYCO    C4   14    -0.039200     12.01000
    15   c3     1   MYCO    C5   15    -0.090400     12.01000
    16   c3     1   MYCO    C6   16    -0.045200     12.01000
    17   c3     1   MYCO    C7   17    -0.048200     12.01000
    18   c2     1   MYCO    C8   18    -0.113400     12.01000
    19   c3     1   MYCO    C9   19    -0.072400     12.01000
    20   c2     1   MYCO   C10   20    -0.173200     12.01000
    21   c3     1   MYCO   C11   21    -0.090100     12.01000
    22   c3     1   MYCO   C12   22    -0.099100     12.01000
    23   c2     1   MYCO   C13   23    -0.101400     12.01000
    24   c3     1   MYCO   C14   24    -0.129400     12.01000
    25   c3     1   MYCO   C15   25    -0.021500     12.01000
    26   c2     1   MYCO   C16   26    -0.186200     12.01000
    27   c3     1   MYCO   C17   27     0.147100     12.01000
    28    c     1   MYCO   C18   28     0.632101     12.01000
    29   c3     1   MYCO   C19   29    -0.071900     12.01000
    30   c3     1   MYCO   C20   30    -0.151400     12.01000
    31   c3     1   MYCO   C21   31    -0.063900     12.01000
    32   c3     1   MYCO   C22   32    -0.075100     12.01000
    33   c3     1   MYCO   C23   33     0.138100     12.01000
    34    c     1   MYCO   C24   34     0.634299     12.01000
    35   c3     1   MYCO   C25   35    -0.095100     12.01000
    36   ce     1   MYCO   C26   36    -0.216200     12.01000
    37   cf     1   MYCO   C27   37    -0.043000     12.01000
    38   cf     1   MYCO   C28   38    -0.095200     12.01000
    39   c3     1   MYCO   C29   39     0.113100     12.01000
    40   c3     1   MYCO   C30   40    -0.107400     12.01000
    41   c3     1   MYCO   C31   41     0.141300     12.01000
    42   c3     1   MYCO   C32   42     0.150100     12.01000
    43   c3     1   MYCO   C33   43    -0.062900     12.01000
    44   ce     1   MYCO   C34   44    -0.102000     12.01000
    45   c2     1   MYCO   C35   45    -0.196200     12.01000
    46   c3     1   MYCO   C36   46    -0.131100     12.01000
    47   ce     1   MYCO   C37   47    -0.073200     12.01000
    48   ce     1   MYCO   C38   48    -0.060200     12.01000
    49   cf     1   MYCO   C39   49    -0.131000     12.01000
    50   ce     1   MYCO   C40   50    -0.124000     12.01000
    51   c3     1   MYCO   C41   51    -0.061900     12.01000
    52   c3     1   MYCO   C42   52    -0.075900     12.01000
    53   cf     1   MYCO   C43   53    -0.123000     12.01000
    54   hc     1   MYCO     H   54     0.063700      1.00800
    55   hc     1   MYCO    H1   55     0.051700      1.00800
    56   h1     1   MYCO    H2   56     0.075700      1.00800
    57   h1     1   MYCO    H3   57     0.088700      1.00800
    58   hc     1   MYCO    H4   58     0.046700      1.00800
    59   hc     1   MYCO    H5   59     0.046700      1.00800
    60   hc     1   MYCO    H6   60     0.059200      1.00800
    61   hc     1   MYCO    H7   61     0.059200      1.00800
    62   hc     1   MYCO    H8   62     0.053200      1.00800
    63   hc     1   MYCO    H9   63     0.053200      1.00800
```

```
    64   hc      1   MYCO   H10   64     0.060200      1.00800
    65   hc      1   MYCO   H11   65     0.060200      1.00800
    66   hc      1   MYCO   H12   66     0.058700      1.00800
    67   hc      1   MYCO   H13   67     0.058700      1.00800
    68   ha      1   MYCO   H14   68     0.120000      1.00800
    69   hc      1   MYCO   H15   69     0.037700      1.00800
    70   hc      1   MYCO   H16   70     0.037700      1.00800
    71   hc      1   MYCO   H17   71     0.037700      1.00800
    72   hc      1   MYCO   H18   72     0.042367      1.00800
    73   hc      1   MYCO   H19   73     0.042367      1.00800
    74   hc      1   MYCO   H20   74     0.042367      1.00800
    75   hc      1   MYCO   H21   75     0.075200      1.00800
    76   hc      1   MYCO   H22   76     0.075200      1.00800
    77   hc      1   MYCO   H23   77     0.050700      1.00800
    78   ha      1   MYCO   H24   78     0.123000      1.00800
    79   h1      1   MYCO   H25   79     0.038700      1.00800
    80   hc      1   MYCO   H26   80     0.049033      1.00800
    81   hc      1   MYCO   H27   81     0.049033      1.00800
    82   hc      1   MYCO   H28   82     0.049033      1.00800
    83   hc      1   MYCO   H29   83     0.058700      1.00800
    84   hc      1   MYCO   H30   84     0.058700      1.00800
    85   hc      1   MYCO   H31   85     0.045700      1.00800
    86   hc      1   MYCO   H32   86     0.045700      1.00800
    87   hc      1   MYCO   H33   87     0.045700      1.00800
    88   hc      1   MYCO   H34   88     0.040700      1.00800
    89   hc      1   MYCO   H35   89     0.040700      1.00800
    90   hc      1   MYCO   H36   90     0.040700      1.00800
    91   h1      1   MYCO   H37   91     0.030700      1.00800
    92   ho      1   MYCO   H38   92     0.397000      1.00800
    93   hc      1   MYCO   H39   93     0.046367      1.00800
    94   hc      1   MYCO   H40   94     0.046367      1.00800
    95   hc      1   MYCO   H41   95     0.046367      1.00800
    96   ha      1   MYCO   H42   96     0.156000      1.00800
    97   ho      1   MYCO   H43   97     0.400000      1.00800
    98   ha      1   MYCO   H44   98     0.148000      1.00800
    99   h1      1   MYCO   H45   99     0.059700      1.00800
   100   hc      1   MYCO   H46  100     0.059200      1.00800
   101   hc      1   MYCO   H47  101     0.059200      1.00800
   102   h1      1   MYCO   H48  102     0.086700      1.00800
   103   h1      1   MYCO   H49  103     0.067700      1.00800
   104   hc      1   MYCO   H50  104     0.048700      1.00800
   105   hc      1   MYCO   H51  105     0.048700      1.00800
   106   hc      1   MYCO   H52  106     0.048700      1.00800
   107   ha      1   MYCO   H53  107     0.133000      1.00800
   108   ha      1   MYCO   H54  108     0.127000      1.00800
   109   hc      1   MYCO   H55  109     0.046700      1.00800
   110   hc      1   MYCO   H56  110     0.046700      1.00800
   111   hc      1   MYCO   H57  111     0.046700      1.00800
   112   ho      1   MYCO   H58  112     0.407000      1.00800
   113   ho      1   MYCO   H59  113     0.401000      1.00800
   114   ho      1   MYCO   H60  114     0.408000      1.00800
   115   ha      1   MYCO   H61  115     0.127000      1.00800
   116   ha      1   MYCO   H62  116     0.124000      1.00800
   117   hc      1   MYCO   H63  117     0.052367      1.00800
   118   hc      1   MYCO   H64  118     0.052367      1.00800
   119   hc      1   MYCO   H65  119     0.052367      1.00800
   120   hc      1   MYCO   H66  120     0.047700      1.00800
   121   hc      1   MYCO   H67  121     0.047700      1.00800
   122   hc      1   MYCO   H68  122     0.047700      1.00800
   123   ha      1   MYCO   H69  123     0.128000      1.00800

[ bonds ]
;   ai     aj funct   r             k
     1     12   1    1.4390e-01    2.5230e+05 ;      O - C2
     1     28   1    1.3430e-01    3.4418e+05 ;      O - C18
     2     13   1    1.4390e-01    2.5230e+05 ;     O1 - C3
     2     34   1    1.3430e-01    3.4418e+05 ;     O1 - C24
     3     27   1    1.4260e-01    2.6284e+05 ;     O2 - C17
     3     92   1    9.7400e-02    3.0928e+05 ;     O2 - H38
     4     28   1    1.2140e-01    5.4225e+05 ;     O3 - C18
     5     33   1    1.4260e-01    2.6284e+05 ;     O4 - C23
     5     97   1    9.7400e-02    3.0928e+05 ;     O4 - H43
     6     34   1    1.2140e-01    5.4225e+05 ;     O5 - C24
```

7	39	1	1.4260e-01	2.6284e+05 ;	O6 - C29
7	112	1	9.7400e-02	3.0928e+05 ;	O6 - H58
8	41	1	1.4260e-01	2.6284e+05 ;	O7 - C31
8	113	1	9.7400e-02	3.0928e+05 ;	O7 - H59
9	42	1	1.4260e-01	2.6284e+05 ;	O8 - C32
9	114	1	9.7400e-02	3.0928e+05 ;	O8 - H60
10	13	1	1.5350e-01	2.5363e+05 ;	C - C3
10	14	1	1.5350e-01	2.5363e+05 ;	C - C4
10	22	1	1.5350e-01	2.5363e+05 ;	C - C12
10	55	1	1.0920e-01	2.8225e+05 ;	C - H1
11	12	1	1.5350e-01	2.5363e+05 ;	C1 - C2
11	16	1	1.5350e-01	2.5363e+05 ;	C1 - C6
11	21	1	1.5350e-01	2.5363e+05 ;	C1 - C11
11	54	1	1.0920e-01	2.8225e+05 ;	C1 - H
12	17	1	1.5350e-01	2.5363e+05 ;	C2 - C7
12	56	1	1.0930e-01	2.8108e+05 ;	C2 - H2
13	15	1	1.5350e-01	2.5363e+05 ;	C3 - C5
13	57	1	1.0930e-01	2.8108e+05 ;	C3 - H3
14	18	1	1.5080e-01	2.7472e+05 ;	C4 - C8
14	58	1	1.0920e-01	2.8225e+05 ;	C4 - H4
14	59	1	1.0920e-01	2.8225e+05 ;	C4 - H5
15	19	1	1.5350e-01	2.5363e+05 ;	C5 - C9
15	60	1	1.0920e-01	2.8225e+05 ;	C5 - H6
15	61	1	1.0920e-01	2.8225e+05 ;	C5 - H7
16	23	1	1.5080e-01	2.7472e+05 ;	C6 - C13
16	62	1	1.0920e-01	2.8225e+05 ;	C6 - H8
16	63	1	1.0920e-01	2.8225e+05 ;	C6 - H9
17	20	1	1.5080e-01	2.7472e+05 ;	C7 - C10
17	64	1	1.0920e-01	2.8225e+05 ;	C7 - H10
17	65	1	1.0920e-01	2.8225e+05 ;	C7 - H11
18	20	1	1.3240e-01	4.9346e+05 ;	C8 - C10
18	29	1	1.5080e-01	2.7472e+05 ;	C8 - C19
19	24	1	1.5350e-01	2.5363e+05 ;	C9 - C14
19	66	1	1.0920e-01	2.8225e+05 ;	C9 - H12
19	67	1	1.0920e-01	2.8225e+05 ;	C9 - H13
20	68	1	1.0870e-01	2.8811e+05 ;	C10 - H14
21	69	1	1.0920e-01	2.8225e+05 ;	C11 - H15
21	70	1	1.0920e-01	2.8225e+05 ;	C11 - H16
21	71	1	1.0920e-01	2.8225e+05 ;	C11 - H17
22	72	1	1.0920e-01	2.8225e+05 ;	C12 - H18
22	73	1	1.0920e-01	2.8225e+05 ;	C12 - H19
22	74	1	1.0920e-01	2.8225e+05 ;	C12 - H20
23	26	1	1.3240e-01	4.9346e+05 ;	C13 - C16
23	31	1	1.5080e-01	2.7472e+05 ;	C13 - C21
24	28	1	1.5080e-01	2.7472e+05 ;	C14 - C18
24	75	1	1.0920e-01	2.8225e+05 ;	C14 - H21
24	76	1	1.0920e-01	2.8225e+05 ;	C14 - H22
25	26	1	1.5080e-01	2.7472e+05 ;	C15 - C16
25	27	1	1.5350e-01	2.5363e+05 ;	C15 - C17
25	32	1	1.5350e-01	2.5363e+05 ;	C15 - C22
25	77	1	1.0920e-01	2.8225e+05 ;	C15 - H23
26	78	1	1.0870e-01	2.8811e+05 ;	C16 - H24
27	30	1	1.5350e-01	2.5363e+05 ;	C17 - C20
27	79	1	1.0930e-01	2.8108e+05 ;	C17 - H25
29	80	1	1.0920e-01	2.8225e+05 ;	C19 - H26
29	81	1	1.0920e-01	2.8225e+05 ;	C19 - H27
29	82	1	1.0920e-01	2.8225e+05 ;	C19 - H28
30	33	1	1.5350e-01	2.5363e+05 ;	C20 - C23
30	83	1	1.0920e-01	2.8225e+05 ;	C20 - H29
30	84	1	1.0920e-01	2.8225e+05 ;	C20 - H30
31	85	1	1.0920e-01	2.8225e+05 ;	C21 - H31
31	86	1	1.0920e-01	2.8225e+05 ;	C21 - H32
31	87	1	1.0920e-01	2.8225e+05 ;	C21 - H33
32	88	1	1.0920e-01	2.8225e+05 ;	C22 - H34
32	89	1	1.0920e-01	2.8225e+05 ;	C22 - H35
32	90	1	1.0920e-01	2.8225e+05 ;	C22 - H36
33	35	1	1.5350e-01	2.5363e+05 ;	C23 - C25
33	91	1	1.0930e-01	2.8108e+05 ;	C23 - H37
34	36	1	1.4740e-01	3.0443e+05 ;	C24 - C26
35	93	1	1.0920e-01	2.8225e+05 ;	C25 - H39
35	94	1	1.0920e-01	2.8225e+05 ;	C25 - H40
35	95	1	1.0920e-01	2.8225e+05 ;	C25 - H41
36	37	1	1.3380e-01	4.7062e+05 ;	C26 - C27

```
    36     96   1    1.0890e-01    2.8577e+05 ;    C26 - H42
    37     38   1    1.4510e-01    3.2677e+05 ;    C27 - C28
    37     98   1    1.0890e-01    2.8577e+05 ;    C27 - H44
    38     43   1    1.5050e-01    2.7723e+05 ;    C28 - C33
    38     44   1    1.3380e-01    4.7062e+05 ;    C28 - C34
    39     40   1    1.5350e-01    2.5363e+05 ;    C29 - C30
    39     41   1    1.5350e-01    2.5363e+05 ;    C29 - C31
    39     99   1    1.0930e-01    2.8108e+05 ;    C29 - H45
    40     42   1    1.5350e-01    2.5363e+05 ;    C30 - C32
    40    100   1    1.0920e-01    2.8225e+05 ;    C30 - H46
    40    101   1    1.0920e-01    2.8225e+05 ;    C30 - H47
    41     45   1    1.5080e-01    2.7472e+05 ;    C31 - C35
    41    102   1    1.0930e-01    2.8108e+05 ;    C31 - H48
    42     46   1    1.5350e-01    2.5363e+05 ;    C32 - C36
    42    103   1    1.0930e-01    2.8108e+05 ;    C32 - H49
    43    104   1    1.0920e-01    2.8225e+05 ;    C33 - H50
    43    105   1    1.0920e-01    2.8225e+05 ;    C33 - H51
    43    106   1    1.0920e-01    2.8225e+05 ;    C33 - H52
    44     47   1    1.4510e-01    3.2677e+05 ;    C34 - C37
    44    107   1    1.0890e-01    2.8577e+05 ;    C34 - H53
    45     48   1    1.3390e-01    4.6903e+05 ;    C35 - C38
    45    108   1    1.0870e-01    2.8811e+05 ;    C35 - H54
    46    109   1    1.0920e-01    2.8225e+05 ;    C36 - H55
    46    110   1    1.0920e-01    2.8225e+05 ;    C36 - H56
    46    111   1    1.0920e-01    2.8225e+05 ;    C36 - H57
    47     49   1    1.3380e-01    4.7062e+05 ;    C37 - C39
    47     51   1    1.5050e-01    2.7723e+05 ;    C37 - C41
    48     50   1    1.4510e-01    3.2677e+05 ;    C38 - C40
    48     52   1    1.5050e-01    2.7723e+05 ;    C38 - C42
    49     53   1    1.4510e-01    3.2677e+05 ;    C39 - C43
    49    115   1    1.0890e-01    2.8577e+05 ;    C39 - H61
    50     53   1    1.3380e-01    4.7062e+05 ;    C40 - C43
    50    116   1    1.0890e-01    2.8577e+05 ;    C40 - H62
    51    120   1    1.0920e-01    2.8225e+05 ;    C41 - H66
    51    121   1    1.0920e-01    2.8225e+05 ;    C41 - H67
    51    122   1    1.0920e-01    2.8225e+05 ;    C41 - H68
    52    117   1    1.0920e-01    2.8225e+05 ;    C42 - H63
    52    118   1    1.0920e-01    2.8225e+05 ;    C42 - H64
    52    119   1    1.0920e-01    2.8225e+05 ;    C42 - H65
    53    123   1    1.0890e-01    2.8577e+05 ;    C43 - H69

[ pairs ]
;   ai     aj    funct
     1     16      1 ;      O - C6
     1     19      1 ;      O - C9
     1     20      1 ;      O - C10
     1     21      1 ;      O - C11
     1     54      1 ;      O - H
     1     64      1 ;      O - H10
     1     65      1 ;      O - H11
     1     75      1 ;      O - H21
     1     76      1 ;      O - H22
     2     14      1 ;     O1 - C4
     2     19      1 ;     O1 - C9
     2     22      1 ;     O1 - C12
     2     37      1 ;     O1 - C27
     2     55      1 ;     O1 - H1
     2     60      1 ;     O1 - H6
     2     61      1 ;     O1 - H7
     2     96      1 ;     O1 - H42
     3     26      1 ;     O2 - C16
     3     32      1 ;     O2 - C22
     3     33      1 ;     O2 - C23
     3     77      1 ;     O2 - H23
     3     83      1 ;     O2 - H29
     3     84      1 ;     O2 - H30
     4     19      1 ;     O3 - C9
     4     75      1 ;     O3 - H21
     4     76      1 ;     O3 - H22
     5     27      1 ;     O4 - C17
     5     83      1 ;     O4 - H29
     5     84      1 ;     O4 - H30
     5     93      1 ;     O4 - H39
```

```
    5     94       1 ;     O4 - H40
    5     95       1 ;     O4 - H41
    6     13       1 ;     O5 - C3
    6     37       1 ;     O5 - C27
    6     96       1 ;     O5 - H42
    7      8       1 ;     O6 - O7
    7     42       1 ;     O6 - C32
    7     45       1 ;     O6 - C35
    7    100       1 ;     O6 - H46
    7    101       1 ;     O6 - H47
    7    102       1 ;     O6 - H48
    8     40       1 ;     O7 - C30
    8     48       1 ;     O7 - C38
    8     99       1 ;     O7 - H45
    8    108       1 ;     O7 - H54
    9     39       1 ;     O8 - C29
    9    100       1 ;     O8 - H46
    9    101       1 ;     O8 - H47
    9    109       1 ;     O8 - H55
    9    110       1 ;     O8 - H56
    9    111       1 ;     O8 - H57
   10     19       1 ;      C - C9
   10     20       1 ;      C - C10
   10     29       1 ;      C - C19
   10     34       1 ;      C - C24
   10     60       1 ;      C - H6
   10     61       1 ;      C - H7
   11     20       1 ;     C1 - C10
   11     26       1 ;     C1 - C16
   11     31       1 ;     C1 - C21
   11     64       1 ;     C1 - H10
   11     65       1 ;     C1 - H11
   12      4       1 ;     C2 - O3
   12     18       1 ;     C2 - C8
   12     23       1 ;     C2 - C13
   12     24       1 ;     C2 - C14
   12     62       1 ;     C2 - H8
   12     63       1 ;     C2 - H9
   12     68       1 ;     C2 - H14
   12     69       1 ;     C2 - H15
   12     70       1 ;     C2 - H16
   12     71       1 ;     C2 - H17
   13     18       1 ;     C3 - C8
   13     24       1 ;     C3 - C14
   13     36       1 ;     C3 - C26
   13     58       1 ;     C3 - H4
   13     59       1 ;     C3 - H5
   13     66       1 ;     C3 - H12
   13     67       1 ;     C3 - H13
   13     72       1 ;     C3 - H18
   13     73       1 ;     C3 - H19
   13     74       1 ;     C3 - H20
   14     15       1 ;     C4 - C5
   14     17       1 ;     C4 - C7
   14     57       1 ;     C4 - H3
   14     68       1 ;     C4 - H14
   14     72       1 ;     C4 - H18
   14     73       1 ;     C4 - H19
   14     74       1 ;     C4 - H20
   14     80       1 ;     C4 - H26
   14     81       1 ;     C4 - H27
   14     82       1 ;     C4 - H28
   15     22       1 ;     C5 - C12
   15     28       1 ;     C5 - C18
   15     34       1 ;     C5 - C24
   15     55       1 ;     C5 - H1
   15     75       1 ;     C5 - H21
   15     76       1 ;     C5 - H22
   16     17       1 ;     C6 - C7
   16     25       1 ;     C6 - C15
   16     56       1 ;     C6 - H2
   16     69       1 ;     C6 - H15
   16     70       1 ;     C6 - H16
```

16	71	1 ;	C6 – H17
16	78	1 ;	C6 – H24
16	85	1 ;	C6 – H31
16	86	1 ;	C6 – H32
16	87	1 ;	C6 – H33
17	21	1 ;	C7 – C11
17	29	1 ;	C7 – C19
17	54	1 ;	C7 – H
18	22	1 ;	C8 – C12
18	55	1 ;	C8 – H1
18	64	1 ;	C8 – H10
18	65	1 ;	C8 – H11
19	57	1 ;	C9 – H3
20	56	1 ;	C10 – H2
20	58	1 ;	C10 – H4
20	59	1 ;	C10 – H5
20	80	1 ;	C10 – H26
20	81	1 ;	C10 – H27
20	82	1 ;	C10 – H28
21	23	1 ;	C11 – C13
21	56	1 ;	C11 – H2
21	62	1 ;	C11 – H8
21	63	1 ;	C11 – H9
22	57	1 ;	C12 – H3
22	58	1 ;	C12 – H4
22	59	1 ;	C12 – H5
23	27	1 ;	C13 – C17
23	32	1 ;	C13 – C22
23	54	1 ;	C13 – H
23	77	1 ;	C13 – H23
24	60	1 ;	C14 – H6
24	61	1 ;	C14 – H7
25	31	1 ;	C15 – C21
25	33	1 ;	C15 – C23
25	83	1 ;	C15 – H29
25	84	1 ;	C15 – H30
25	92	1 ;	C15 – H38
26	30	1 ;	C16 – C20
26	62	1 ;	C16 – H8
26	63	1 ;	C16 – H9
26	79	1 ;	C16 – H25
26	85	1 ;	C16 – H31
26	86	1 ;	C16 – H32
26	87	1 ;	C16 – H33
26	88	1 ;	C16 – H34
26	89	1 ;	C16 – H35
26	90	1 ;	C16 – H36
27	35	1 ;	C17 – C25
27	78	1 ;	C17 – H24
27	88	1 ;	C17 – H34
27	89	1 ;	C17 – H35
27	90	1 ;	C17 – H36
27	91	1 ;	C17 – H37
28	11	1 ;	C18 – C1
28	17	1 ;	C18 – C7
28	56	1 ;	C18 – H2
28	66	1 ;	C18 – H12
28	67	1 ;	C18 – H13
29	58	1 ;	C19 – H4
29	59	1 ;	C19 – H5
29	68	1 ;	C19 – H14
30	32	1 ;	C20 – C22
30	77	1 ;	C20 – H23
30	92	1 ;	C20 – H38
30	93	1 ;	C20 – H39
30	94	1 ;	C20 – H40
30	95	1 ;	C20 – H41
30	97	1 ;	C20 – H43
31	62	1 ;	C21 – H8
31	63	1 ;	C21 – H9
31	78	1 ;	C21 – H24
32	78	1 ;	C22 – H24
32	79	1 ;	C22 – H25

33	79	1 ;	C23 – H25
34	38	1 ;	C24 – C28
34	57	1 ;	C24 – H3
34	98	1 ;	C24 – H44
35	83	1 ;	C25 – H29
35	84	1 ;	C25 – H30
35	97	1 ;	C25 – H43
36	43	1 ;	C26 – C33
36	44	1 ;	C26 – C34
37	47	1 ;	C27 – C37
37	104	1 ;	C27 – H50
37	105	1 ;	C27 – H51
37	106	1 ;	C27 – H52
37	107	1 ;	C27 – H53
38	49	1 ;	C28 – C39
38	51	1 ;	C28 – C41
38	96	1 ;	C28 – H42
39	46	1 ;	C29 – C36
39	48	1 ;	C29 – C38
39	103	1 ;	C29 – H49
39	108	1 ;	C29 – H54
39	113	1 ;	C29 – H59
40	45	1 ;	C30 – C35
40	102	1 ;	C30 – H48
40	109	1 ;	C30 – H55
40	110	1 ;	C30 – H56
40	111	1 ;	C30 – H57
40	112	1 ;	C30 – H58
40	114	1 ;	C30 – H60
41	42	1 ;	C31 – C32
41	50	1 ;	C31 – C40
41	52	1 ;	C31 – C42
41	100	1 ;	C31 – H46
41	101	1 ;	C31 – H47
41	112	1 ;	C31 – H58
42	99	1 ;	C32 – H45
43	47	1 ;	C33 – C37
43	98	1 ;	C33 – H44
43	107	1 ;	C33 – H53
44	53	1 ;	C34 – C43
44	98	1 ;	C34 – H44
44	104	1 ;	C34 – H50
44	105	1 ;	C34 – H51
44	106	1 ;	C34 – H52
44	115	1 ;	C34 – H61
44	120	1 ;	C34 – H66
44	121	1 ;	C34 – H67
44	122	1 ;	C34 – H68
45	53	1 ;	C35 – C43
45	99	1 ;	C35 – H45
45	113	1 ;	C35 – H59
45	116	1 ;	C35 – H62
45	117	1 ;	C35 – H63
45	118	1 ;	C35 – H64
45	119	1 ;	C35 – H65
46	100	1 ;	C36 – H46
46	101	1 ;	C36 – H47
46	114	1 ;	C36 – H60
47	50	1 ;	C37 – C40
47	123	1 ;	C37 – H69
48	49	1 ;	C38 – C39
48	102	1 ;	C38 – H48
48	123	1 ;	C38 – H69
49	107	1 ;	C39 – H53
49	116	1 ;	C39 – H62
49	120	1 ;	C39 – H66
49	121	1 ;	C39 – H67
49	122	1 ;	C39 – H68
50	108	1 ;	C40 – H54
50	115	1 ;	C40 – H61
50	117	1 ;	C40 – H63
50	118	1 ;	C40 – H64
50	119	1 ;	C40 – H65

```
    51    53     1 ;    C41 - C43
    51   107     1 ;    C41 - H53
    51   115     1 ;    C41 - H61
    52    53     1 ;    C42 - C43
    52   108     1 ;    C42 - H54
    52   116     1 ;    C42 - H62
    54    56     1 ;      H - H2
    54    62     1 ;      H - H8
    54    63     1 ;      H - H9
    54    69     1 ;      H - H15
    54    70     1 ;      H - H16
    54    71     1 ;      H - H17
    55    57     1 ;     H1 - H3
    55    58     1 ;     H1 - H4
    55    59     1 ;     H1 - H5
    55    72     1 ;     H1 - H18
    55    73     1 ;     H1 - H19
    55    74     1 ;     H1 - H20
    56    64     1 ;     H2 - H10
    56    65     1 ;     H2 - H11
    57    60     1 ;     H3 - H6
    57    61     1 ;     H3 - H7
    60    66     1 ;     H6 - H12
    60    67     1 ;     H6 - H13
    61    66     1 ;     H7 - H12
    61    67     1 ;     H7 - H13
    64    68     1 ;    H10 - H14
    65    68     1 ;    H11 - H14
    66    75     1 ;    H12 - H21
    66    76     1 ;    H12 - H22
    67    75     1 ;    H13 - H21
    67    76     1 ;    H13 - H22
    77    78     1 ;    H23 - H24
    77    79     1 ;    H23 - H25
    77    88     1 ;    H23 - H34
    77    89     1 ;    H23 - H35
    77    90     1 ;    H23 - H36
    79    83     1 ;    H25 - H29
    79    84     1 ;    H25 - H30
    79    92     1 ;    H25 - H38
    83    91     1 ;    H29 - H37
    84    91     1 ;    H30 - H37
    91    93     1 ;    H37 - H39
    91    94     1 ;    H37 - H40
    91    95     1 ;    H37 - H41
    91    97     1 ;    H37 - H43
    96    98     1 ;    H42 - H44
    99   100     1 ;    H45 - H46
    99   101     1 ;    H45 - H47
    99   102     1 ;    H45 - H48
    99   112     1 ;    H45 - H58
   100   103     1 ;    H46 - H49
   101   103     1 ;    H47 - H49
   102   108     1 ;    H48 - H54
   102   113     1 ;    H48 - H59
   103   109     1 ;    H49 - H55
   103   110     1 ;    H49 - H56
   103   111     1 ;    H49 - H57
   103   114     1 ;    H49 - H60
   115   123     1 ;    H61 - H69
   116   123     1 ;    H62 - H69

 [ angles ]
;   ai     aj     ak    funct   theta         cth
     1     12     11      1    1.0842e+02    5.6718e+02 ;      O - C2     - C1
     1     12     17      1    1.0842e+02    5.6718e+02 ;      O - C2     - C7
     1     12     56      1    1.0882e+02    4.2543e+02 ;      O - C2     - H2
     1     28      4      1    1.2333e+02    6.3538e+02 ;      O - C18    - O3
     1     28     24      1    1.1196e+02    5.7957e+02 ;      O - C18    - C14
     2     13     10      1    1.0842e+02    5.6718e+02 ;     O1 - C3     - C
     2     13     15      1    1.0842e+02    5.6718e+02 ;     O1 - C3     - C5
     2     13     57      1    1.0882e+02    4.2543e+02 ;     O1 - C3     - H3
     2     34      6      1    1.2333e+02    6.3538e+02 ;     O1 - C24    - O5
```

2	34	36	1	1.1262e+02	5.8618e+02	;	O1	-	C24	-	C26
3	27	25	1	1.0943e+02	5.6668e+02	;	O2	-	C17	-	C15
3	27	30	1	1.0943e+02	5.6668e+02	;	O2	-	C17	-	C20
3	27	79	1	1.0988e+02	4.2652e+02	;	O2	-	C17	-	H25
4	28	24	1	1.2311e+02	5.6928e+02	;	O3	-	C18	-	C14
5	33	30	1	1.0943e+02	5.6668e+02	;	O4	-	C23	-	C20
5	33	35	1	1.0943e+02	5.6668e+02	;	O4	-	C23	-	C25
5	33	91	1	1.0988e+02	4.2652e+02	;	O4	-	C23	-	H37
6	34	36	1	1.2292e+02	5.7965e+02	;	O5	-	C24	-	C26
7	39	40	1	1.0943e+02	5.6668e+02	;	O6	-	C29	-	C30
7	39	41	1	1.0943e+02	5.6668e+02	;	O6	-	C29	-	C31
7	39	99	1	1.0988e+02	4.2652e+02	;	O6	-	C29	-	H45
8	41	39	1	1.0943e+02	5.6668e+02	;	O7	-	C31	-	C29
8	41	45	1	1.1021e+02	5.7053e+02	;	O7	-	C31	-	C35
8	41	102	1	1.0988e+02	4.2652e+02	;	O7	-	C31	-	H48
9	42	40	1	1.0943e+02	5.6668e+02	;	O8	-	C32	-	C30
9	42	46	1	1.0943e+02	5.6668e+02	;	O8	-	C32	-	C36
9	42	103	1	1.0988e+02	4.2652e+02	;	O8	-	C32	-	H49
10	13	15	1	1.1063e+02	5.2894e+02	;	C	-	C3	-	C5
10	13	57	1	1.1007e+02	3.8794e+02	;	C	-	C3	-	H3
10	14	18	1	1.1144e+02	5.3162e+02	;	C	-	C4	-	C8
10	14	58	1	1.1005e+02	3.8802e+02	;	C	-	C4	-	H4
10	14	59	1	1.1005e+02	3.8802e+02	;	C	-	C4	-	H5
10	22	72	1	1.1005e+02	3.8802e+02	;	C	-	C12	-	H18
10	22	73	1	1.1005e+02	3.8802e+02	;	C	-	C12	-	H19
10	22	74	1	1.1005e+02	3.8802e+02	;	C	-	C12	-	H20
11	12	17	1	1.1063e+02	5.2894e+02	;	C1	-	C2	-	C7
11	12	56	1	1.1007e+02	3.8794e+02	;	C1	-	C2	-	H2
11	16	23	1	1.1144e+02	5.3162e+02	;	C1	-	C6	-	C13
11	16	62	1	1.1005e+02	3.8802e+02	;	C1	-	C6	-	H8
11	16	63	1	1.1005e+02	3.8802e+02	;	C1	-	C6	-	H9
11	21	69	1	1.1005e+02	3.8802e+02	;	C1	-	C11	-	H15
11	21	70	1	1.1005e+02	3.8802e+02	;	C1	-	C11	-	H16
11	21	71	1	1.1005e+02	3.8802e+02	;	C1	-	C11	-	H17
12	1	28	1	1.1514e+02	5.3246e+02	;	C2	-	O	-	C18
12	11	16	1	1.1063e+02	5.2894e+02	;	C2	-	C1	-	C6
12	11	21	1	1.1063e+02	5.2894e+02	;	C2	-	C1	-	C11
12	11	54	1	1.1005e+02	3.8802e+02	;	C2	-	C1	-	H
12	17	20	1	1.1144e+02	5.3162e+02	;	C2	-	C7	-	C10
12	17	64	1	1.1005e+02	3.8802e+02	;	C2	-	C7	-	H10
12	17	65	1	1.1005e+02	3.8802e+02	;	C2	-	C7	-	H11
13	2	34	1	1.1514e+02	5.3246e+02	;	C3	-	O1	-	C24
13	10	14	1	1.1063e+02	5.2894e+02	;	C3	-	C	-	C4
13	10	22	1	1.1063e+02	5.2894e+02	;	C3	-	C	-	C12
13	10	55	1	1.1005e+02	3.8802e+02	;	C3	-	C	-	H1
13	15	19	1	1.1063e+02	5.2894e+02	;	C3	-	C5	-	C9
13	15	60	1	1.1005e+02	3.8802e+02	;	C3	-	C5	-	H6
13	15	61	1	1.1005e+02	3.8802e+02	;	C3	-	C5	-	H7
14	10	22	1	1.1063e+02	5.2894e+02	;	C4	-	C	-	C12
14	10	55	1	1.1005e+02	3.8802e+02	;	C4	-	C	-	H1
14	18	20	1	1.2342e+02	5.3831e+02	;	C4	-	C8	-	C10
14	18	29	1	1.1652e+02	5.2467e+02	;	C4	-	C8	-	C19
15	13	57	1	1.1007e+02	3.8794e+02	;	C5	-	C3	-	H3
15	19	24	1	1.1063e+02	5.2894e+02	;	C5	-	C9	-	C14
15	19	66	1	1.1005e+02	3.8802e+02	;	C5	-	C9	-	H12
15	19	67	1	1.1005e+02	3.8802e+02	;	C5	-	C9	-	H13
16	11	21	1	1.1063e+02	5.2894e+02	;	C6	-	C1	-	C11
16	11	54	1	1.1005e+02	3.8802e+02	;	C6	-	C1	-	H
16	23	26	1	1.2342e+02	5.3831e+02	;	C6	-	C13	-	C16
16	23	31	1	1.1652e+02	5.2467e+02	;	C6	-	C13	-	C21
17	12	56	1	1.1007e+02	3.8794e+02	;	C7	-	C2	-	H2
17	20	18	1	1.2342e+02	5.3831e+02	;	C7	-	C10	-	C8
17	20	68	1	1.1730e+02	3.8208e+02	;	C7	-	C10	-	H14
18	14	58	1	1.1049e+02	3.9355e+02	;	C8	-	C4	-	H4
18	14	59	1	1.1049e+02	3.9355e+02	;	C8	-	C4	-	H5
18	20	68	1	1.2094e+02	4.1873e+02	;	C8	-	C10	-	H14
18	29	80	1	1.1049e+02	3.9355e+02	;	C8	-	C19	-	H26
18	29	81	1	1.1049e+02	3.9355e+02	;	C8	-	C19	-	H27
18	29	82	1	1.1049e+02	3.9355e+02	;	C8	-	C19	-	H28
19	15	60	1	1.1005e+02	3.8802e+02	;	C9	-	C5	-	H6
19	15	61	1	1.1005e+02	3.8802e+02	;	C9	-	C5	-	H7
19	24	28	1	1.1053e+02	5.3379e+02	;	C9	-	C14	-	C18
19	24	75	1	1.1005e+02	3.8802e+02	;	C9	-	C14	-	H21

19	24	76	1	1.1005e+02	3.8802e+02	;	C9 - C14	- H22
20	17	64	1	1.1049e+02	3.9355e+02	;	C10 - C7	- H10
20	17	65	1	1.1049e+02	3.9355e+02	;	C10 - C7	- H11
20	18	29	1	1.2342e+02	5.3831e+02	;	C10 - C8	- C19
21	11	54	1	1.1005e+02	3.8802e+02	;	C11 - C1	- H
22	10	55	1	1.1005e+02	3.8802e+02	;	C12 - C	- H1
23	16	62	1	1.1049e+02	3.9355e+02	;	C13 - C6	- H8
23	16	63	1	1.1049e+02	3.9355e+02	;	C13 - C6	- H9
23	26	25	1	1.2342e+02	5.3831e+02	;	C13 - C16	- C15
23	26	78	1	1.2094e+02	4.1873e+02	;	C13 - C16	- H24
23	31	85	1	1.1049e+02	3.9355e+02	;	C13 - C21	- H31
23	31	86	1	1.1049e+02	3.9355e+02	;	C13 - C21	- H32
23	31	87	1	1.1049e+02	3.9355e+02	;	C13 - C21	- H33
24	19	66	1	1.1005e+02	3.8802e+02	;	C14 - C9	- H12
24	19	67	1	1.1005e+02	3.8802e+02	;	C14 - C9	- H13
25	26	78	1	1.1730e+02	3.8208e+02	;	C15 - C16	- H24
25	27	30	1	1.1063e+02	5.2894e+02	;	C15 - C17	- C20
25	27	79	1	1.1007e+02	3.8794e+02	;	C15 - C17	- H25
25	32	88	1	1.1005e+02	3.8802e+02	;	C15 - C22	- H34
25	32	89	1	1.1005e+02	3.8802e+02	;	C15 - C22	- H35
25	32	90	1	1.1005e+02	3.8802e+02	;	C15 - C22	- H36
26	23	31	1	1.2342e+02	5.3831e+02	;	C16 - C13	- C21
26	25	27	1	1.1144e+02	5.3162e+02	;	C16 - C15	- C17
26	25	32	1	1.1144e+02	5.3162e+02	;	C16 - C15	- C22
26	25	77	1	1.1049e+02	3.9355e+02	;	C16 - C15	- H23
27	3	92	1	1.0816e+02	3.9405e+02	;	C17 - O2	- H38
27	25	32	1	1.1063e+02	5.2894e+02	;	C17 - C15	- C22
27	25	77	1	1.1005e+02	3.8802e+02	;	C17 - C15	- H23
27	30	33	1	1.1063e+02	5.2894e+02	;	C17 - C20	- C23
27	30	83	1	1.1005e+02	3.8802e+02	;	C17 - C20	- H29
27	30	84	1	1.1005e+02	3.8802e+02	;	C17 - C20	- H30
28	24	75	1	1.0968e+02	3.9497e+02	;	C18 - C14	- H21
28	24	76	1	1.0968e+02	3.9497e+02	;	C18 - C14	- H22
30	27	79	1	1.1007e+02	3.8794e+02	;	C20 - C17	- H25
30	33	35	1	1.1063e+02	5.2894e+02	;	C20 - C23	- C25
30	33	91	1	1.1007e+02	3.8794e+02	;	C20 - C23	- H37
32	25	77	1	1.1005e+02	3.8802e+02	;	C22 - C15	- H23
33	5	97	1	1.0816e+02	3.9405e+02	;	C23 - O4	- H43
33	30	83	1	1.1005e+02	3.8802e+02	;	C23 - C20	- H29
33	30	84	1	1.1005e+02	3.8802e+02	;	C23 - C20	- H30
33	35	93	1	1.1005e+02	3.8802e+02	;	C23 - C25	- H39
33	35	94	1	1.1005e+02	3.8802e+02	;	C23 - C25	- H40
33	35	95	1	1.1005e+02	3.8802e+02	;	C23 - C25	- H41
34	36	37	1	1.2641e+02	5.3773e+02	;	C24 - C26	- C27
34	36	96	1	1.1726e+02	3.8987e+02	;	C24 - C26	- H42
35	33	91	1	1.1007e+02	3.8794e+02	;	C25 - C23	- H37
36	37	38	1	1.2433e+02	5.4743e+02	;	C26 - C27	- C28
36	37	98	1	1.1798e+02	4.2041e+02	;	C26 - C27	- H44
37	36	96	1	1.1829e+02	4.1991e+02	;	C27 - C26	- H42
37	38	43	1	1.1801e+02	5.3154e+02	;	C27 - C28	- C33
37	38	44	1	1.2433e+02	5.4743e+02	;	C27 - C28	- C34
38	37	98	1	1.1590e+02	3.9748e+02	;	C28 - C27	- H44
38	43	104	1	1.1098e+02	3.9330e+02	;	C28 - C33	- H50
38	43	105	1	1.1098e+02	3.9330e+02	;	C28 - C33	- H51
38	43	106	1	1.1098e+02	3.9330e+02	;	C28 - C33	- H52
38	44	47	1	1.2405e+02	5.4802e+02	;	C28 - C34	- C37
38	44	107	1	1.1829e+02	4.1991e+02	;	C28 - C34	- H53
39	7	112	1	1.0816e+02	3.9405e+02	;	C29 - O6	- H58
39	40	42	1	1.1063e+02	5.2894e+02	;	C29 - C30	- C32
39	40	100	1	1.1005e+02	3.8802e+02	;	C29 - C30	- H46
39	40	101	1	1.1005e+02	3.8802e+02	;	C29 - C30	- H47
39	41	45	1	1.1144e+02	5.3162e+02	;	C29 - C31	- C35
39	41	102	1	1.1007e+02	3.8794e+02	;	C29 - C31	- H48
40	39	41	1	1.1063e+02	5.2894e+02	;	C30 - C29	- C31
40	39	99	1	1.1007e+02	3.8794e+02	;	C30 - C29	- H45
40	42	46	1	1.1063e+02	5.2894e+02	;	C30 - C32	- C36
40	42	103	1	1.1007e+02	3.8794e+02	;	C30 - C32	- H49
41	8	113	1	1.0816e+02	3.9405e+02	;	C31 - O7	- H59
41	39	99	1	1.1007e+02	3.8794e+02	;	C31 - C29	- H45
41	45	48	1	1.2302e+02	5.3714e+02	;	C31 - C35	- C38
41	45	108	1	1.1730e+02	3.8208e+02	;	C31 - C35	- H54
42	9	114	1	1.0816e+02	3.9405e+02	;	C32 - O8	- H60
42	40	100	1	1.1005e+02	3.8802e+02	;	C32 - C30	- H46

```
    42    40   101     1    1.1005e+02    3.8802e+02 ;    C32 - C30    - H47
    42    46   109     1    1.1005e+02    3.8802e+02 ;    C32 - C36    - H55
    42    46   110     1    1.1005e+02    3.8802e+02 ;    C32 - C36    - H56
    42    46   111     1    1.1005e+02    3.8802e+02 ;    C32 - C36    - H57
    43    38    44     1    1.2203e+02    5.4015e+02 ;    C33 - C28    - C34
    44    47    49     1    1.2405e+02    5.4802e+02 ;    C34 - C37    - C39
    44    47    51     1    1.1659e+02    5.3480e+02 ;    C34 - C37    - C41
    45    41   102     1    1.1046e+02    3.9355e+02 ;    C35 - C31    - H48
    45    48    50     1    1.2308e+02    5.5003e+02 ;    C35 - C38    - C40
    45    48    52     1    1.2289e+02    5.3806e+02 ;    C35 - C38    - C42
    46    42   103     1    1.1007e+02    3.8794e+02 ;    C36 - C32    - H49
    47    44   107     1    1.1590e+02    3.9748e+02 ;    C37 - C34    - H53
    47    49    53     1    1.2433e+02    5.4743e+02 ;    C37 - C39    - C43
    47    49   115     1    1.1798e+02    4.2041e+02 ;    C37 - C39    - H61
    47    51   120     1    1.1098e+02    3.9330e+02 ;    C37 - C41    - H66
    47    51   121     1    1.1098e+02    3.9330e+02 ;    C37 - C41    - H67
    47    51   122     1    1.1098e+02    3.9330e+02 ;    C37 - C41    - H68
    48    45   108     1    1.2119e+02    4.1480e+02 ;    C38 - C35    - H54
    48    50    53     1    1.2405e+02    5.4802e+02 ;    C38 - C40    - C43
    48    50   116     1    1.1590e+02    3.9748e+02 ;    C38 - C40    - H62
    48    52   117     1    1.1098e+02    3.9330e+02 ;    C38 - C42    - H63
    48    52   118     1    1.1098e+02    3.9330e+02 ;    C38 - C42    - H64
    48    52   119     1    1.1098e+02    3.9330e+02 ;    C38 - C42    - H65
    49    47    51     1    1.2218e+02    5.3982e+02 ;    C39 - C37    - C41
    49    53    50     1    1.2433e+02    5.4743e+02 ;    C39 - C43    - C40
    49    53   123     1    1.1590e+02    3.9748e+02 ;    C39 - C43    - H69
    50    48    52     1    1.1659e+02    5.3480e+02 ;    C40 - C38    - C42
    50    53   123     1    1.1798e+02    4.2041e+02 ;    C40 - C43    - H69
    53    49   115     1    1.1590e+02    3.9748e+02 ;    C43 - C39    - H61
    53    50   116     1    1.1829e+02    4.1991e+02 ;    C43 - C40    - H62
    58    14    59     1    1.0835e+02    3.2995e+02 ;     H4 - C4     - H5
    60    15    61     1    1.0835e+02    3.2995e+02 ;     H6 - C5     - H7
    62    16    63     1    1.0835e+02    3.2995e+02 ;     H8 - C6     - H9
    64    17    65     1    1.0835e+02    3.2995e+02 ;    H10 - C7     - H11
    66    19    67     1    1.0835e+02    3.2995e+02 ;    H12 - C9     - H13
    69    21    70     1    1.0835e+02    3.2995e+02 ;    H15 - C11    - H16
    69    21    71     1    1.0835e+02    3.2995e+02 ;    H15 - C11    - H17
    70    21    71     1    1.0835e+02    3.2995e+02 ;    H16 - C11    - H17
    72    22    73     1    1.0835e+02    3.2995e+02 ;    H18 - C12    - H19
    72    22    74     1    1.0835e+02    3.2995e+02 ;    H18 - C12    - H20
    73    22    74     1    1.0835e+02    3.2995e+02 ;    H19 - C12    - H20
    75    24    76     1    1.0835e+02    3.2995e+02 ;    H21 - C14    - H22
    80    29    81     1    1.0835e+02    3.2995e+02 ;    H26 - C19    - H27
    80    29    82     1    1.0835e+02    3.2995e+02 ;    H26 - C19    - H28
    81    29    82     1    1.0835e+02    3.2995e+02 ;    H27 - C19    - H28
    83    30    84     1    1.0835e+02    3.2995e+02 ;    H29 - C20    - H30
    85    31    86     1    1.0835e+02    3.2995e+02 ;    H31 - C21    - H32
    85    31    87     1    1.0835e+02    3.2995e+02 ;    H31 - C21    - H33
    86    31    87     1    1.0835e+02    3.2995e+02 ;    H32 - C21    - H33
    88    32    89     1    1.0835e+02    3.2995e+02 ;    H34 - C22    - H35
    88    32    90     1    1.0835e+02    3.2995e+02 ;    H34 - C22    - H36
    89    32    90     1    1.0835e+02    3.2995e+02 ;    H35 - C22    - H36
    93    35    94     1    1.0835e+02    3.2995e+02 ;    H39 - C25    - H40
    93    35    95     1    1.0835e+02    3.2995e+02 ;    H39 - C25    - H41
    94    35    95     1    1.0835e+02    3.2995e+02 ;    H40 - C25    - H41
   100    40   101     1    1.0835e+02    3.2995e+02 ;    H46 - C30    - H47
   104    43   105     1    1.0835e+02    3.2995e+02 ;    H50 - C33    - H51
   104    43   106     1    1.0835e+02    3.2995e+02 ;    H50 - C33    - H52
   105    43   106     1    1.0835e+02    3.2995e+02 ;    H51 - C33    - H52
   109    46   110     1    1.0835e+02    3.2995e+02 ;    H55 - C36    - H56
   109    46   111     1    1.0835e+02    3.2995e+02 ;    H55 - C36    - H57
   110    46   111     1    1.0835e+02    3.2995e+02 ;    H56 - C36    - H57
   117    52   118     1    1.0835e+02    3.2995e+02 ;    H63 - C42    - H64
   117    52   119     1    1.0835e+02    3.2995e+02 ;    H63 - C42    - H65
   118    52   119     1    1.0835e+02    3.2995e+02 ;    H64 - C42    - H65
   120    51   121     1    1.0835e+02    3.2995e+02 ;    H66 - C41    - H67
   120    51   122     1    1.0835e+02    3.2995e+02 ;    H66 - C41    - H68
   121    51   122     1    1.0835e+02    3.2995e+02 ;    H67 - C41    - H68

[ dihedrals ] ; propers
; for gromacs 4.5 or higher, using funct 9
;    i      j      k      l   func   phase     kd      pn
     1     12     11     16      9     0.00   0.65084   3 ;      O-    C2-    C1-    C6
```

1	12	11	21	9	0.00	0.65084	3	;	O-	C2-	C1-	C11
1	12	11	54	9	0.00	0.00000	0	;	O-	C2-	C1-	H
1	12	11	54	9	0.00	1.04600	1	;	O-	C2-	C1-	H
1	12	17	20	9	0.00	0.65084	3	;	O-	C2-	C7-	C10
1	12	17	64	9	0.00	0.00000	0	;	O-	C2-	C7-	H10
1	12	17	64	9	0.00	1.04600	1	;	O-	C2-	C7-	H10
1	12	17	65	9	0.00	0.00000	0	;	O-	C2-	C7-	H11
1	12	17	65	9	0.00	1.04600	1	;	O-	C2-	C7-	H11
1	28	24	19	9	180.00	0.00000	2	;	O-	C18-	C14-	C9
1	28	24	75	9	180.00	0.00000	2	;	O-	C18-	C14-	H21
1	28	24	76	9	180.00	0.00000	2	;	O-	C18-	C14-	H22
2	13	10	14	9	0.00	0.65084	3	;	O1-	C3-	C-	C4
2	13	10	22	9	0.00	0.65084	3	;	O1-	C3-	C-	C12
2	13	10	55	9	0.00	0.00000	0	;	O1-	C3-	C-	H1
2	13	10	55	9	0.00	1.04600	1	;	O1-	C3-	C-	H1
2	13	15	19	9	0.00	0.65084	3	;	O1-	C3-	C5-	C9
2	13	15	60	9	0.00	0.00000	0	;	O1-	C3-	C5-	H6
2	13	15	60	9	0.00	1.04600	1	;	O1-	C3-	C5-	H6
2	13	15	61	9	0.00	0.00000	0	;	O1-	C3-	C5-	H7
2	13	15	61	9	0.00	1.04600	1	;	O1-	C3-	C5-	H7
2	34	36	37	9	180.00	9.10020	2	;	O1-	C24-	C26-	C27
2	34	36	96	9	180.00	9.10020	2	;	O1-	C24-	C26-	H42
3	27	25	26	9	0.00	0.65084	3	;	O2-	C17-	C15-	C16
3	27	25	32	9	0.00	0.65084	3	;	O2-	C17-	C15-	C22
3	27	25	77	9	0.00	0.00000	0	;	O2-	C17-	C15-	H23
3	27	25	77	9	0.00	1.04600	1	;	O2-	C17-	C15-	H23
3	27	30	33	9	0.00	0.65084	3	;	O2-	C17-	C20-	C23
3	27	30	83	9	0.00	0.00000	0	;	O2-	C17-	C20-	H29
3	27	30	83	9	0.00	1.04600	1	;	O2-	C17-	C20-	H29
3	27	30	84	9	0.00	0.00000	0	;	O2-	C17-	C20-	H30
3	27	30	84	9	0.00	1.04600	1	;	O2-	C17-	C20-	H30
4	28	24	19	9	180.00	0.00000	2	;	O3-	C18-	C14-	C9
4	28	24	75	9	0.00	3.34720	1	;	O3-	C18-	C14-	H21
4	28	24	75	9	180.00	0.33472	3	;	O3-	C18-	C14-	H21
4	28	24	76	9	0.00	3.34720	1	;	O3-	C18-	C14-	H22
4	28	24	76	9	180.00	0.33472	3	;	O3-	C18-	C14-	H22
5	33	30	27	9	0.00	0.65084	3	;	O4-	C23-	C20-	C17
5	33	30	83	9	0.00	0.00000	0	;	O4-	C23-	C20-	H29
5	33	30	83	9	0.00	1.04600	1	;	O4-	C23-	C20-	H29
5	33	30	84	9	0.00	0.00000	0	;	O4-	C23-	C20-	H30
5	33	30	84	9	0.00	1.04600	1	;	O4-	C23-	C20-	H30
5	33	35	93	9	0.00	0.00000	0	;	O4-	C23-	C25-	H39
5	33	35	93	9	0.00	1.04600	1	;	O4-	C23-	C25-	H39
5	33	35	94	9	0.00	0.00000	0	;	O4-	C23-	C25-	H40
5	33	35	94	9	0.00	1.04600	1	;	O4-	C23-	C25-	H40
5	33	35	95	9	0.00	0.00000	0	;	O4-	C23-	C25-	H41
5	33	35	95	9	0.00	1.04600	1	;	O4-	C23-	C25-	H41
6	34	2	13	9	180.00	5.85760	1	;	O5-	C24-	O1-	C3
6	34	2	13	9	180.00	11.29680	2	;	O5-	C24-	O1-	C3
6	34	36	37	9	180.00	9.10020	2	;	O5-	C24-	C26-	C27
6	34	36	96	9	180.00	9.10020	2	;	O5-	C24-	C26-	H42
7	39	40	42	9	0.00	0.65084	3	;	O6-	C29-	C30-	C32
7	39	40	100	9	0.00	0.00000	0	;	O6-	C29-	C30-	H46
7	39	40	100	9	0.00	1.04600	1	;	O6-	C29-	C30-	H46
7	39	40	101	9	0.00	0.00000	0	;	O6-	C29-	C30-	H47
7	39	40	101	9	0.00	1.04600	1	;	O6-	C29-	C30-	H47
7	39	41	8	9	0.00	0.60250	3	;	O6-	C29-	C31-	O7
7	39	41	8	9	0.00	4.91620	2	;	O6-	C29-	C31-	O7
7	39	41	45	9	0.00	0.65084	3	;	O6-	C29-	C31-	C35
7	39	41	102	9	0.00	0.00000	0	;	O6-	C29-	C31-	H48
7	39	41	102	9	0.00	1.04600	1	;	O6-	C29-	C31-	H48
8	41	39	40	9	0.00	0.65084	3	;	O7-	C31-	C29-	C30
8	41	39	99	9	0.00	0.00000	0	;	O7-	C31-	C29-	H45
8	41	39	99	9	0.00	1.04600	1	;	O7-	C31-	C29-	H45
8	41	45	48	9	0.00	0.00000	0	;	O7-	C31-	C35-	C38
8	41	45	108	9	0.00	0.00000	0	;	O7-	C31-	C35-	H54
9	42	40	39	9	0.00	0.65084	3	;	O8-	C32-	C30-	C29
9	42	40	100	9	0.00	0.00000	0	;	O8-	C32-	C30-	H46
9	42	40	100	9	0.00	1.04600	1	;	O8-	C32-	C30-	H46
9	42	40	101	9	0.00	0.00000	0	;	O8-	C32-	C30-	H47
9	42	40	101	9	0.00	1.04600	1	;	O8-	C32-	C30-	H47
9	42	46	109	9	0.00	0.00000	0	;	O8-	C32-	C36-	H55
9	42	46	109	9	0.00	1.04600	1	;	O8-	C32-	C36-	H55

9	42	46	110	9	0.00	0.00000	0	;	O8-	C32-	C36-	H56
9	42	46	110	9	0.00	1.04600	1	;	O8-	C32-	C36-	H56
9	42	46	111	9	0.00	0.00000	0	;	O8-	C32-	C36-	H57
9	42	46	111	9	0.00	1.04600	1	;	O8-	C32-	C36-	H57
10	13	2	34	9	0.00	1.60247	3	;	C-	C3-	O1-	C24
10	13	2	34	9	180.00	3.34720	1	;	C-	C3-	O1-	C24
10	13	15	19	9	0.00	0.75312	3	;	C-	C3-	C5-	C9
10	13	15	19	9	180.00	0.83680	1	;	C-	C3-	C5-	C9
10	13	15	19	9	180.00	1.04600	2	;	C-	C3-	C5-	C9
10	13	15	60	9	0.00	0.66944	3	;	C-	C3-	C5-	H6
10	13	15	61	9	0.00	0.66944	3	;	C-	C3-	C5-	H7
10	14	18	20	9	0.00	0.00000	0	;	C-	C4-	C8-	C10
10	14	18	29	9	0.00	0.00000	0	;	C-	C4-	C8-	C19
11	12	17	20	9	0.00	0.65084	3	;	C1-	C2-	C7-	C10
11	12	17	64	9	0.00	0.66944	3	;	C1-	C2-	C7-	H10
11	12	17	65	9	0.00	0.66944	3	;	C1-	C2-	C7-	H11
11	16	23	26	9	0.00	0.00000	0	;	C1-	C6-	C13-	C16
11	16	23	31	9	0.00	0.00000	0	;	C1-	C6-	C13-	C21
12	1	28	4	9	180.00	5.85760	1	;	C2-	O-	C18-	O3
12	1	28	4	9	180.00	11.29680	2	;	C2-	O-	C18-	O3
12	1	28	24	9	180.00	11.29680	2	;	C2-	O-	C18-	C14
12	11	16	23	9	0.00	0.65084	3	;	C2-	C1-	C6-	C13
12	11	16	62	9	0.00	0.66944	3	;	C2-	C1-	C6-	H8
12	11	16	63	9	0.00	0.66944	3	;	C2-	C1-	C6-	H9
12	11	21	69	9	0.00	0.66944	3	;	C2-	C1-	C11-	H15
12	11	21	70	9	0.00	0.66944	3	;	C2-	C1-	C11-	H16
12	11	21	71	9	0.00	0.66944	3	;	C2-	C1-	C11-	H17
12	17	20	18	9	0.00	0.00000	0	;	C2-	C7-	C10-	C8
12	17	20	68	9	0.00	0.00000	0	;	C2-	C7-	C10-	H14
13	2	34	36	9	180.00	11.29680	2	;	C3-	O1-	C24-	C26
13	10	14	18	9	0.00	0.65084	3	;	C3-	C-	C4-	C8
13	10	14	58	9	0.00	0.66944	3	;	C3-	C-	C4-	H4
13	10	14	59	9	0.00	0.66944	3	;	C3-	C-	C4-	H5
13	10	22	72	9	0.00	0.66944	3	;	C3-	C-	C12-	H18
13	10	22	73	9	0.00	0.66944	3	;	C3-	C-	C12-	H19
13	10	22	74	9	0.00	0.66944	3	;	C3-	C-	C12-	H20
13	15	19	24	9	0.00	0.75312	3	;	C3-	C5-	C9-	C14
13	15	19	24	9	180.00	0.83680	1	;	C3-	C5-	C9-	C14
13	15	19	24	9	180.00	1.04600	2	;	C3-	C5-	C9-	C14
13	15	19	66	9	0.00	0.66944	3	;	C3-	C5-	C9-	H12
13	15	19	67	9	0.00	0.66944	3	;	C3-	C5-	C9-	H13
14	10	13	15	9	0.00	0.75312	3	;	C4-	C-	C3-	C5
14	10	13	15	9	180.00	0.83680	1	;	C4-	C-	C3-	C5
14	10	13	15	9	180.00	1.04600	2	;	C4-	C-	C3-	C5
14	10	13	57	9	0.00	0.65084	3	;	C4-	C-	C3-	H3
14	10	22	72	9	0.00	0.66944	3	;	C4-	C-	C12-	H18
14	10	22	73	9	0.00	0.66944	3	;	C4-	C-	C12-	H19
14	10	22	74	9	0.00	0.66944	3	;	C4-	C-	C12-	H20
14	18	20	17	9	180.00	7.94960	1	;	C4-	C8-	C10-	C7
14	18	20	17	9	180.00	27.82360	2	;	C4-	C8-	C10-	C7
14	18	20	68	9	180.00	27.82360	2	;	C4-	C8-	C10-	H14
14	18	29	80	9	0.00	0.00000	0	;	C4-	C8-	C19-	H26
14	18	29	81	9	0.00	0.00000	0	;	C4-	C8-	C19-	H27
14	18	29	82	9	0.00	0.00000	0	;	C4-	C8-	C19-	H28
15	13	2	34	9	0.00	1.60247	3	;	C5-	C3-	O1-	C24
15	13	2	34	9	180.00	3.34720	1	;	C5-	C3-	O1-	C24
15	13	10	22	9	0.00	0.75312	3	;	C5-	C3-	C-	C12
15	13	10	22	9	180.00	0.83680	1	;	C5-	C3-	C-	C12
15	13	10	22	9	180.00	1.04600	2	;	C5-	C3-	C-	C12
15	13	10	55	9	0.00	0.66944	3	;	C5-	C3-	C-	H1
15	19	24	28	9	0.00	0.65084	3	;	C5-	C9-	C14-	C18
15	19	24	75	9	0.00	0.66944	3	;	C5-	C9-	C14-	H21
15	19	24	76	9	0.00	0.66944	3	;	C5-	C9-	C14-	H22
16	11	12	17	9	0.00	0.75312	3	;	C6-	C1-	C2-	C7
16	11	12	17	9	180.00	0.83680	1	;	C6-	C1-	C2-	C7
16	11	12	17	9	180.00	1.04600	2	;	C6-	C1-	C2-	C7
16	11	12	56	9	0.00	0.65084	3	;	C6-	C1-	C2-	H2
16	11	21	69	9	0.00	0.66944	3	;	C6-	C1-	C11-	H15
16	11	21	70	9	0.00	0.66944	3	;	C6-	C1-	C11-	H16
16	11	21	71	9	0.00	0.66944	3	;	C6-	C1-	C11-	H17
16	23	26	25	9	180.00	7.94960	1	;	C6-	C13-	C16-	C15
16	23	26	25	9	180.00	27.82360	2	;	C6-	C13-	C16-	C15
16	23	26	78	9	180.00	27.82360	2	;	C6-	C13-	C16-	H24

16	23	31	85	9	0.00	0.00000	0	;	C6-	C13-	C21-	H31
16	23	31	86	9	0.00	0.00000	0	;	C6-	C13-	C21-	H32
16	23	31	87	9	0.00	0.00000	0	;	C6-	C13-	C21-	H33
17	12	11	21	9	0.00	0.75312	3	;	C7-	C2-	C1-	C11
17	12	11	21	9	180.00	0.83680	1	;	C7-	C2-	C1-	C11
17	12	11	21	9	180.00	1.04600	2	;	C7-	C2-	C1-	C11
17	12	11	54	9	0.00	0.66944	3	;	C7-	C2-	C1-	H
17	20	18	29	9	180.00	7.94960	1	;	C7-	C10-	C8-	C19
17	20	18	29	9	180.00	27.82360	2	;	C7-	C10-	C8-	C19
18	14	10	22	9	0.00	0.65084	3	;	C8-	C4-	C-	C12
18	14	10	55	9	0.00	0.65084	3	;	C8-	C4-	C-	H1
18	20	17	64	9	0.00	4.81160	1	;	C8-	C10-	C7-	H10
18	20	17	64	9	180.00	1.58992	3	;	C8-	C10-	C7-	H10
18	20	17	65	9	0.00	4.81160	1	;	C8-	C10-	C7-	H11
18	20	17	65	9	180.00	1.58992	3	;	C8-	C10-	C7-	H11
19	15	13	57	9	0.00	0.65084	3	;	C9-	C5-	C3-	H3
20	17	12	56	9	0.00	0.65084	3	;	C10-	C7-	C2-	H2
20	18	14	58	9	0.00	4.81160	1	;	C10-	C8-	C4-	H4
20	18	14	58	9	180.00	1.58992	3	;	C10-	C8-	C4-	H4
20	18	14	59	9	0.00	4.81160	1	;	C10-	C8-	C4-	H5
20	18	14	59	9	180.00	1.58992	3	;	C10-	C8-	C4-	H5
20	18	29	80	9	0.00	4.81160	1	;	C10-	C8-	C19-	H26
20	18	29	80	9	180.00	1.58992	3	;	C10-	C8-	C19-	H26
20	18	29	81	9	0.00	4.81160	1	;	C10-	C8-	C19-	H27
20	18	29	81	9	180.00	1.58992	3	;	C10-	C8-	C19-	H27
20	18	29	82	9	0.00	4.81160	1	;	C10-	C8-	C19-	H28
20	18	29	82	9	180.00	1.58992	3	;	C10-	C8-	C19-	H28
21	11	12	56	9	0.00	0.65084	3	;	C11-	C1-	C2-	H2
21	11	16	23	9	0.00	0.65084	3	;	C11-	C1-	C6-	C13
21	11	16	62	9	0.00	0.66944	3	;	C11-	C1-	C6-	H8
21	11	16	63	9	0.00	0.66944	3	;	C11-	C1-	C6-	H9
22	10	13	57	9	0.00	0.65084	3	;	C12-	C-	C3-	H3
22	10	14	58	9	0.00	0.66944	3	;	C12-	C-	C4-	H4
22	10	14	59	9	0.00	0.66944	3	;	C12-	C-	C4-	H5
23	16	11	54	9	0.00	0.65084	3	;	C13-	C6-	C1-	H
23	26	25	27	9	0.00	0.00000	0	;	C13-	C16-	C15-	C17
23	26	25	32	9	0.00	0.00000	0	;	C13-	C16-	C15-	C22
23	26	25	77	9	0.00	4.81160	1	;	C13-	C16-	C15-	H23
23	26	25	77	9	180.00	1.58992	3	;	C13-	C16-	C15-	H23
24	19	15	60	9	0.00	0.66944	3	;	C14-	C9-	C5-	H6
24	19	15	61	9	0.00	0.66944	3	;	C14-	C9-	C5-	H7
25	26	23	31	9	180.00	7.94960	1	;	C15-	C16-	C13-	C21
25	26	23	31	9	180.00	27.82360	2	;	C15-	C16-	C13-	C21
25	27	3	92	9	0.00	0.66944	3	;	C15-	C17-	O2-	H38
25	27	3	92	9	0.00	1.04600	1	;	C15-	C17-	O2-	H38
25	27	30	33	9	0.00	0.75312	3	;	C15-	C17-	C20-	C23
25	27	30	33	9	180.00	0.83680	1	;	C15-	C17-	C20-	C23
25	27	30	33	9	180.00	1.04600	2	;	C15-	C17-	C20-	C23
25	27	30	83	9	0.00	0.66944	3	;	C15-	C17-	C20-	H29
25	27	30	84	9	0.00	0.66944	3	;	C15-	C17-	C20-	H30
26	23	16	62	9	0.00	4.81160	1	;	C16-	C13-	C6-	H8
26	23	16	62	9	180.00	1.58992	3	;	C16-	C13-	C6-	H8
26	23	16	63	9	0.00	4.81160	1	;	C16-	C13-	C6-	H9
26	23	16	63	9	180.00	1.58992	3	;	C16-	C13-	C6-	H9
26	23	31	85	9	0.00	4.81160	1	;	C16-	C13-	C21-	H31
26	23	31	85	9	180.00	1.58992	3	;	C16-	C13-	C21-	H31
26	23	31	86	9	0.00	4.81160	1	;	C16-	C13-	C21-	H32
26	23	31	86	9	180.00	1.58992	3	;	C16-	C13-	C21-	H32
26	23	31	87	9	0.00	4.81160	1	;	C16-	C13-	C21-	H33
26	23	31	87	9	180.00	1.58992	3	;	C16-	C13-	C21-	H33
26	25	27	30	9	0.00	0.65084	3	;	C16-	C15-	C17-	C20
26	25	27	79	9	0.00	0.65084	3	;	C16-	C15-	C17-	H25
26	25	32	88	9	0.00	0.65084	3	;	C16-	C15-	C22-	H34
26	25	32	89	9	0.00	0.65084	3	;	C16-	C15-	C22-	H35
26	25	32	90	9	0.00	0.65084	3	;	C16-	C15-	C22-	H36
27	25	26	78	9	0.00	0.00000	0	;	C17-	C15-	C16-	H24
27	25	32	88	9	0.00	0.66944	3	;	C17-	C15-	C22-	H34
27	25	32	89	9	0.00	0.66944	3	;	C17-	C15-	C22-	H35
27	25	32	90	9	0.00	0.66944	3	;	C17-	C15-	C22-	H36
27	30	33	35	9	0.00	0.75312	3	;	C17-	C20-	C23-	C25
27	30	33	35	9	180.00	0.83680	1	;	C17-	C20-	C23-	C25
27	30	33	35	9	180.00	1.04600	2	;	C17-	C20-	C23-	C25
27	30	33	91	9	0.00	0.65084	3	;	C17-	C20-	C23-	H37

28	1	12	11	9	0.00	1.60247	3	;	C18–	O–	C2–	C1
28	1	12	11	9	180.00	3.34720	1	;	C18–	O–	C2–	C1
28	1	12	17	9	0.00	1.60247	3	;	C18–	O–	C2–	C7
28	1	12	17	9	180.00	3.34720	1	;	C18–	O–	C2–	C7
28	1	12	56	9	0.00	1.60387	3	;	C18–	O–	C2–	H2
28	24	19	66	9	0.00	0.65084	3	;	C18–	C14–	C9–	H12
28	24	19	67	9	0.00	0.65084	3	;	C18–	C14–	C9–	H13
29	18	14	58	9	0.00	0.00000	0	;	C19–	C8–	C4–	H4
29	18	14	59	9	0.00	0.00000	0	;	C19–	C8–	C4–	H5
29	18	20	68	9	180.00	27.82360	2	;	C19–	C8–	C10–	H14
30	27	3	92	9	0.00	0.66944	3	;	C20–	C17–	O2–	H38
30	27	3	92	9	0.00	1.04600	1	;	C20–	C17–	O2–	H38
30	27	25	32	9	0.00	0.75312	3	;	C20–	C17–	C15–	C22
30	27	25	32	9	180.00	0.83680	1	;	C20–	C17–	C15–	C22
30	27	25	32	9	180.00	1.04600	2	;	C20–	C17–	C15–	C22
30	27	25	77	9	0.00	0.66944	3	;	C20–	C17–	C15–	H23
30	33	5	97	9	0.00	0.66944	3	;	C20–	C23–	O4–	H43
30	33	5	97	9	0.00	1.04600	1	;	C20–	C23–	O4–	H43
30	33	35	93	9	0.00	0.66944	3	;	C20–	C23–	C25–	H39
30	33	35	94	9	0.00	0.66944	3	;	C20–	C23–	C25–	H40
30	33	35	95	9	0.00	0.66944	3	;	C20–	C23–	C25–	H41
31	23	16	62	9	0.00	0.00000	0	;	C21–	C13–	C6–	H8
31	23	16	63	9	0.00	0.00000	0	;	C21–	C13–	C6–	H9
31	23	26	78	9	180.00	27.82360	2	;	C21–	C13–	C16–	H24
32	25	26	78	9	0.00	0.00000	0	;	C22–	C15–	C16–	H24
32	25	27	79	9	0.00	0.65084	3	;	C22–	C15–	C17–	H25
33	30	27	79	9	0.00	0.65084	3	;	C23–	C20–	C17–	H25
34	2	13	57	9	0.00	1.60387	3	;	C24–	O1–	C3–	H3
34	36	37	38	9	180.00	27.82360	2	;	C24–	C26–	C27–	C28
34	36	37	98	9	180.00	27.82360	2	;	C24–	C26–	C27–	H44
35	33	5	97	9	0.00	0.66944	3	;	C25–	C23–	O4–	H43
35	33	5	97	9	0.00	1.04600	1	;	C25–	C23–	O4–	H43
35	33	30	83	9	0.00	0.66944	3	;	C25–	C23–	C20–	H29
35	33	30	84	9	0.00	0.66944	3	;	C25–	C23–	C20–	H30
36	37	38	43	9	180.00	4.18400	2	;	C26–	C27–	C28–	C33
36	37	38	44	9	180.00	4.18400	2	;	C26–	C27–	C28–	C34
37	38	43	104	9	0.00	0.00000	0	;	C27–	C28–	C33–	H50
37	38	43	105	9	0.00	0.00000	0	;	C27–	C28–	C33–	H51
37	38	43	106	9	0.00	0.00000	0	;	C27–	C28–	C33–	H52
37	38	44	47	9	180.00	27.82360	2	;	C27–	C28–	C34–	C37
37	38	44	107	9	180.00	27.82360	2	;	C27–	C28–	C34–	H53
38	37	36	96	9	180.00	27.82360	2	;	C28–	C27–	C26–	H42
38	44	47	49	9	180.00	4.18400	2	;	C28–	C34–	C37–	C39
38	44	47	51	9	180.00	4.18400	2	;	C28–	C34–	C37–	C41
39	40	42	46	9	0.00	0.75312	3	;	C29–	C30–	C32–	C36
39	40	42	46	9	180.00	0.83680	1	;	C29–	C30–	C32–	C36
39	40	42	46	9	180.00	1.04600	2	;	C29–	C30–	C32–	C36
39	40	42	103	9	0.00	0.65084	3	;	C29–	C30–	C32–	H49
39	41	8	113	9	0.00	0.66944	3	;	C29–	C31–	O7–	H59
39	41	8	113	9	0.00	1.04600	1	;	C29–	C31–	O7–	H59
39	41	45	48	9	0.00	0.00000	0	;	C29–	C31–	C35–	C38
39	41	45	108	9	0.00	0.00000	0	;	C29–	C31–	C35–	H54
40	39	7	112	9	0.00	0.66944	3	;	C30–	C29–	O6–	H58
40	39	7	112	9	0.00	1.04600	1	;	C30–	C29–	O6–	H58
40	39	41	45	9	0.00	0.65084	3	;	C30–	C29–	C31–	C35
40	39	41	102	9	0.00	0.65084	3	;	C30–	C29–	C31–	H48
40	42	9	114	9	0.00	0.66944	3	;	C30–	C32–	O8–	H60
40	42	9	114	9	0.00	1.04600	1	;	C30–	C32–	O8–	H60
40	42	46	109	9	0.00	0.66944	3	;	C30–	C32–	C36–	H55
40	42	46	110	9	0.00	0.66944	3	;	C30–	C32–	C36–	H56
40	42	46	111	9	0.00	0.66944	3	;	C30–	C32–	C36–	H57
41	39	7	112	9	0.00	0.66944	3	;	C31–	C29–	O6–	H58
41	39	7	112	9	0.00	1.04600	1	;	C31–	C29–	O6–	H58
41	39	40	42	9	0.00	0.75312	3	;	C31–	C29–	C30–	C32
41	39	40	42	9	180.00	0.83680	1	;	C31–	C29–	C30–	C32
41	39	40	42	9	180.00	1.04600	2	;	C31–	C29–	C30–	C32
41	39	40	100	9	0.00	0.66944	3	;	C31–	C29–	C30–	H46
41	39	40	101	9	0.00	0.66944	3	;	C31–	C29–	C30–	H47
41	45	48	50	9	180.00	27.82360	2	;	C31–	C35–	C38–	C40
41	45	48	52	9	180.00	27.82360	2	;	C31–	C35–	C38–	C42
42	40	39	99	9	0.00	0.65084	3	;	C32–	C30–	C29–	H45
43	38	37	98	9	180.00	4.18400	2	;	C33–	C28–	C27–	H44
43	38	44	47	9	180.00	27.82360	2	;	C33–	C28–	C34–	C37

43	38	44	107	9	180.00	27.82360	2	;	C33-	C28-	C34-	H53
44	38	37	98	9	180.00	4.18400	2	;	C34-	C28-	C27-	H44
44	38	43	104	9	0.00	0.00000	0	;	C34-	C28-	C33-	H50
44	38	43	105	9	0.00	0.00000	0	;	C34-	C28-	C33-	H51
44	38	43	106	9	0.00	0.00000	0	;	C34-	C28-	C33-	H52
44	47	49	53	9	180.00	27.82360	2	;	C34-	C37-	C39-	C43
44	47	49	115	9	180.00	27.82360	2	;	C34-	C37-	C39-	H61
44	47	51	120	9	0.00	0.00000	0	;	C34-	C37-	C41-	H66
44	47	51	121	9	0.00	0.00000	0	;	C34-	C37-	C41-	H67
44	47	51	122	9	0.00	0.00000	0	;	C34-	C37-	C41-	H68
45	41	8	113	9	0.00	0.69733	3	;	C35-	C31-	O7-	H59
45	41	39	99	9	0.00	0.65084	3	;	C35-	C31-	C29-	H45
45	48	50	53	9	180.00	4.18400	2	;	C35-	C38-	C40-	C43
45	48	50	116	9	180.00	4.18400	2	;	C35-	C38-	C40-	H62
45	48	52	117	9	0.00	4.81160	1	;	C35-	C38-	C42-	H63
45	48	52	117	9	180.00	1.58992	3	;	C35-	C38-	C42-	H63
45	48	52	118	9	0.00	4.81160	1	;	C35-	C38-	C42-	H64
45	48	52	118	9	180.00	1.58992	3	;	C35-	C38-	C42-	H64
45	48	52	119	9	0.00	4.81160	1	;	C35-	C38-	C42-	H65
45	48	52	119	9	180.00	1.58992	3	;	C35-	C38-	C42-	H65
46	42	9	114	9	0.00	0.66944	3	;	C36-	C32-	O8-	H60
46	42	9	114	9	0.00	1.04600	1	;	C36-	C32-	O8-	H60
46	42	40	100	9	0.00	0.66944	3	;	C36-	C32-	C30-	H46
46	42	40	101	9	0.00	0.66944	3	;	C36-	C32-	C30-	H47
47	49	53	50	9	180.00	4.18400	2	;	C37-	C39-	C43-	C40
47	49	53	123	9	180.00	4.18400	2	;	C37-	C39-	C43-	H69
48	45	41	102	9	0.00	0.00000	0	;	C38-	C35-	C31-	H48
48	50	53	49	9	180.00	27.82360	2	;	C38-	C40-	C43-	C39
48	50	53	123	9	180.00	27.82360	2	;	C38-	C40-	C43-	H69
49	47	44	107	9	180.00	4.18400	2	;	C39-	C37-	C34-	H53
49	47	51	120	9	0.00	4.81160	1	;	C39-	C37-	C41-	H66
49	47	51	120	9	180.00	1.58992	3	;	C39-	C37-	C41-	H66
49	47	51	121	9	0.00	4.81160	1	;	C39-	C37-	C41-	H67
49	47	51	121	9	180.00	1.58992	3	;	C39-	C37-	C41-	H67
49	47	51	122	9	0.00	4.81160	1	;	C39-	C37-	C41-	H68
49	47	51	122	9	180.00	1.58992	3	;	C39-	C37-	C41-	H68
49	53	50	116	9	180.00	27.82360	2	;	C39-	C43-	C40-	H62
50	48	45	108	9	180.00	27.82360	2	;	C40-	C38-	C35-	H54
50	48	52	117	9	0.00	0.00000	0	;	C40-	C38-	C42-	H63
50	48	52	118	9	0.00	0.00000	0	;	C40-	C38-	C42-	H64
50	48	52	119	9	0.00	0.00000	0	;	C40-	C38-	C42-	H65
50	53	49	115	9	180.00	4.18400	2	;	C40-	C43-	C39-	H61
51	47	44	107	9	180.00	4.18400	2	;	C41-	C37-	C34-	H53
51	47	49	53	9	180.00	27.82360	2	;	C41-	C37-	C39-	C43
51	47	49	115	9	180.00	27.82360	2	;	C41-	C37-	C39-	H61
52	48	45	108	9	180.00	27.82360	2	;	C42-	C38-	C35-	H54
52	48	50	53	9	180.00	4.18400	2	;	C42-	C38-	C40-	C43
52	48	50	116	9	180.00	4.18400	2	;	C42-	C38-	C40-	H62
54	11	12	56	9	0.00	0.65084	3	;	H-	C1-	C2-	H2
54	11	16	62	9	0.00	0.62760	3	;	H-	C1-	C6-	H8
54	11	16	63	9	0.00	0.62760	3	;	H-	C1-	C6-	H9
54	11	21	69	9	0.00	0.62760	3	;	H-	C1-	C11-	H15
54	11	21	70	9	0.00	0.62760	3	;	H-	C1-	C11-	H16
54	11	21	71	9	0.00	0.62760	3	;	H-	C1-	C11-	H17
55	10	13	57	9	0.00	0.65084	3	;	H1-	C-	C3-	H3
55	10	14	58	9	0.00	0.62760	3	;	H1-	C-	C4-	H4
55	10	14	59	9	0.00	0.62760	3	;	H1-	C-	C4-	H5
55	10	22	72	9	0.00	0.62760	3	;	H1-	C-	C12-	H18
55	10	22	73	9	0.00	0.62760	3	;	H1-	C-	C12-	H19
55	10	22	74	9	0.00	0.62760	3	;	H1-	C-	C12-	H20
56	12	17	64	9	0.00	0.65084	3	;	H2-	C2-	C7-	H10
56	12	17	65	9	0.00	0.65084	3	;	H2-	C2-	C7-	H11
57	13	15	60	9	0.00	0.65084	3	;	H3-	C3-	C5-	H6
57	13	15	61	9	0.00	0.65084	3	;	H3-	C3-	C5-	H7
60	15	19	66	9	0.00	0.62760	3	;	H6-	C5-	C9-	H12
60	15	19	67	9	0.00	0.62760	3	;	H6-	C5-	C9-	H13
61	15	19	66	9	0.00	0.62760	3	;	H7-	C5-	C9-	H12
61	15	19	67	9	0.00	0.62760	3	;	H7-	C5-	C9-	H13
64	17	20	68	9	0.00	0.00000	0	;	H10-	C7-	C10-	H14
65	17	20	68	9	0.00	0.00000	0	;	H11-	C7-	C10-	H14
66	19	24	75	9	0.00	0.62760	3	;	H12-	C9-	C14-	H21
66	19	24	76	9	0.00	0.62760	3	;	H12-	C9-	C14-	H22
67	19	24	75	9	0.00	0.62760	3	;	H13-	C9-	C14-	H21

```
    67     19     24     76      9     0.00   0.62760   3 ;     H13-    C9-   C14-   H22
    77     25     26     78      9     0.00   0.00000   0 ;     H23-   C15-   C16-   H24
    77     25     27     79      9     0.00   0.65084   3 ;     H23-   C15-   C17-   H25
    77     25     32     88      9     0.00   0.62760   3 ;     H23-   C15-   C22-   H34
    77     25     32     89      9     0.00   0.62760   3 ;     H23-   C15-   C22-   H35
    77     25     32     90      9     0.00   0.62760   3 ;     H23-   C15-   C22-   H36
    79     27      3     92      9     0.00   0.69733   3 ;     H25-   C17-    O2-   H38
    79     27     30     83      9     0.00   0.65084   3 ;     H25-   C17-   C20-   H29
    79     27     30     84      9     0.00   0.65084   3 ;     H25-   C17-   C20-   H30
    83     30     33     91      9     0.00   0.65084   3 ;     H29-   C20-   C23-   H37
    84     30     33     91      9     0.00   0.65084   3 ;     H30-   C20-   C23-   H37
    91     33      5     97      9     0.00   0.69733   3 ;     H37-   C23-    O4-   H43
    91     33     35     93      9     0.00   0.65084   3 ;     H37-   C23-   C25-   H39
    91     33     35     94      9     0.00   0.65084   3 ;     H37-   C23-   C25-   H40
    91     33     35     95      9     0.00   0.65084   3 ;     H37-   C23-   C25-   H41
    96     36     37     98      9   180.00  27.82360   2 ;     H42-   C26-   C27-   H44
    99     39      7    112      9     0.00   0.69733   3 ;     H45-   C29-    O6-   H58
    99     39     40    100      9     0.00   0.65084   3 ;     H45-   C29-   C30-   H46
    99     39     40    101      9     0.00   0.65084   3 ;     H45-   C29-   C30-   H47
    99     39     41    102      9     0.00   0.65084   3 ;     H45-   C29-   C31-   H48
   100     40     42    103      9     0.00   0.65084   3 ;     H46-   C30-   C32-   H49
   101     40     42    103      9     0.00   0.65084   3 ;     H47-   C30-   C32-   H49
   102     41      8    113      9     0.00   0.69733   3 ;     H48-   C31-    O7-   H59
   102     41     45    108      9     0.00   0.00000   0 ;     H48-   C31-   C35-   H54
   103     42      9    114      9     0.00   0.69733   3 ;     H49-   C32-    O8-   H60
   103     42     46    109      9     0.00   0.65084   3 ;     H49-   C32-   C36-   H55
   103     42     46    110      9     0.00   0.65084   3 ;     H49-   C32-   C36-   H56
   103     42     46    111      9     0.00   0.65084   3 ;     H49-   C32-   C36-   H57
   115     49     53    123      9   180.00   4.18400   2 ;     H61-   C39-   C43-   H69
   116     50     53    123      9   180.00  27.82360   2 ;     H62-   C40-   C43-   H69

[ dihedrals ] ; impropers
; treated as propers in GROMACS to use correct AMBER analytical function
;    i      j      k      l   func   phase     kd      pn
     1     28      4     24      4   180.00  43.93200   2 ;      O-   C18-    O3-   C14
    18     17     20     68      4   180.00   4.60240   2 ;     C8-    C7-   C10-   H14
    20     14     18     29      4   180.00   4.60240   2 ;    C10-    C4-    C8-   C19
    23     25     26     78      4   180.00   4.60240   2 ;    C13-   C15-   C16-   H24
    26     16     23     31      4   180.00   4.60240   2 ;    C16-    C6-   C13-   C21
    34     37     36     96      4   180.00   4.60240   2 ;    C24-   C27-   C26-   H42
    36      6     34      2      4   180.00  43.93200   2 ;    C26-    O5-   C24-    O1
    36     38     37     98      4   180.00   4.60240   2 ;    C26-   C28-   C27-   H44
    41     48     45    108      4   180.00   4.60240   2 ;    C31-   C38-   C35-   H54
    43     44     38     37      4   180.00   4.60240   2 ;    C33-   C34-   C28-   C27
    45     52     48     50      4   180.00   4.60240   2 ;    C35-   C42-   C38-   C40
    47     38     44    107      4   180.00   4.60240   2 ;    C37-   C28-   C34-   H53
    47     53     49    115      4   180.00   4.60240   2 ;    C37-   C43-   C39-   H61
    48     53     50    116      4   180.00   4.60240   2 ;    C38-   C43-   C40-   H62
    50     49     53    123      4   180.00   4.60240   2 ;    C40-   C39-   C43-   H69
    51     44     47     49      4   180.00   4.60240   2 ;    C41-   C34-   C37-   C39

Coarse-grained

[ moleculetype ]
; Name            nrexcl
MYCO             1

[ atoms ]
;   nr       type  resnr residue  atom   cgnr     charge       mass  typeB    chargeB      massB
    1          P1    1    MYCO     N1     1         0        72.0000
    2          P1    1    MYCO     N2     2         0        72.0000
    3          C3    1    MYCO     N3     3         0        72.0000
    4          C1    1    MYCO     N4     4         0        72.0000
    5          Na    1    MYCO     N5     5         0        72.0000
    6          C3    1    MYCO     N6     6         0        72.0000
    7          C1    1    MYCO     N7     7         0        72.0000
    8          Na    1    MYCO     N8     8         0        72.0000
    9          C3    1    MYCO     N9     9         0        72.0000
   10          C3    1    MYCO     N10    10        0        72.0000
   11          C3    1    MYCO     N11    11        0        72.0000
   12          P4    1    MYCO     N12    12        0        72.0000
   13          P1    1    MYCO     N13    13        0        72.0000
```

```
[ bonds ]
;  ai    aj funct            c0            c1            c2            c3
#ifdef FLEXIBLE
    4    5   1  0.3000   30000
    4    6   1  0.4000   30000
    5    7   1  0.3700   30000
    9   10   1  0.3350   30000
    8    9   1  0.3850   40000
   10   11   1  0.4100   30000
#endif
    1    2   1  0.3400  15000
    2    3   1  0.3400  15000
    6    7   1  0.3400  20000
    7    8   1  0.3100  5000
   11   12   1  0.3300  10000
   12   13   1  0.3300  10000
    3    4   1  0.3200  15000

[ angles ]
;  ai    aj    ak funct            c0            c1            c2            c3
   1     2     3    2  110.000  50.000
   2     3     4    2  131.000 200.000
   3     4     5    2  105.000  50.000
   3     4     6    2  110.000 100.000
   4     5     7    2  115.000 300.000
   4     6     7    2   90.000 250.000
   5     7     8    2   90.000 200.000;
   6     7     8    2   82.000 300.000;
   7     8     9    2  125.000 500.000
   8     9    10    2  160.000 300.000
   9    10    11    2  134.000 600.000
  10    11    12    2  120.000 200.000;
  11    12    13    2  120.000  25.000

[ dihedrals ]
 4  5  7  8   2 -106.00  100.00
 5  7  8  9   2 -175.00   20.00

#ifndef FLEXIBLE
[ constraints ]
    4    5   1  0.3000   30000
    4    6   1  0.4000   30000
    5    7   1  0.3700   30000
    9   10   1  0.3350   30000
    8    9   1  0.3850   40000
   10   11   1  0.4100   30000
#endif

[ exclusions ]
1 3
5 8
6 8
```
