# Supplementary figures and images for: Membrane perturbing properties of toxin mycolactone from Mycobacterium ulcerans
Source: PLoS Comput Biol. 2018 Feb 5;14(2):e1005972. doi: 10.1371/journal.pcbi.1005972 (PMC5814095; doi:10.1371/journal.pcbi.1005972)

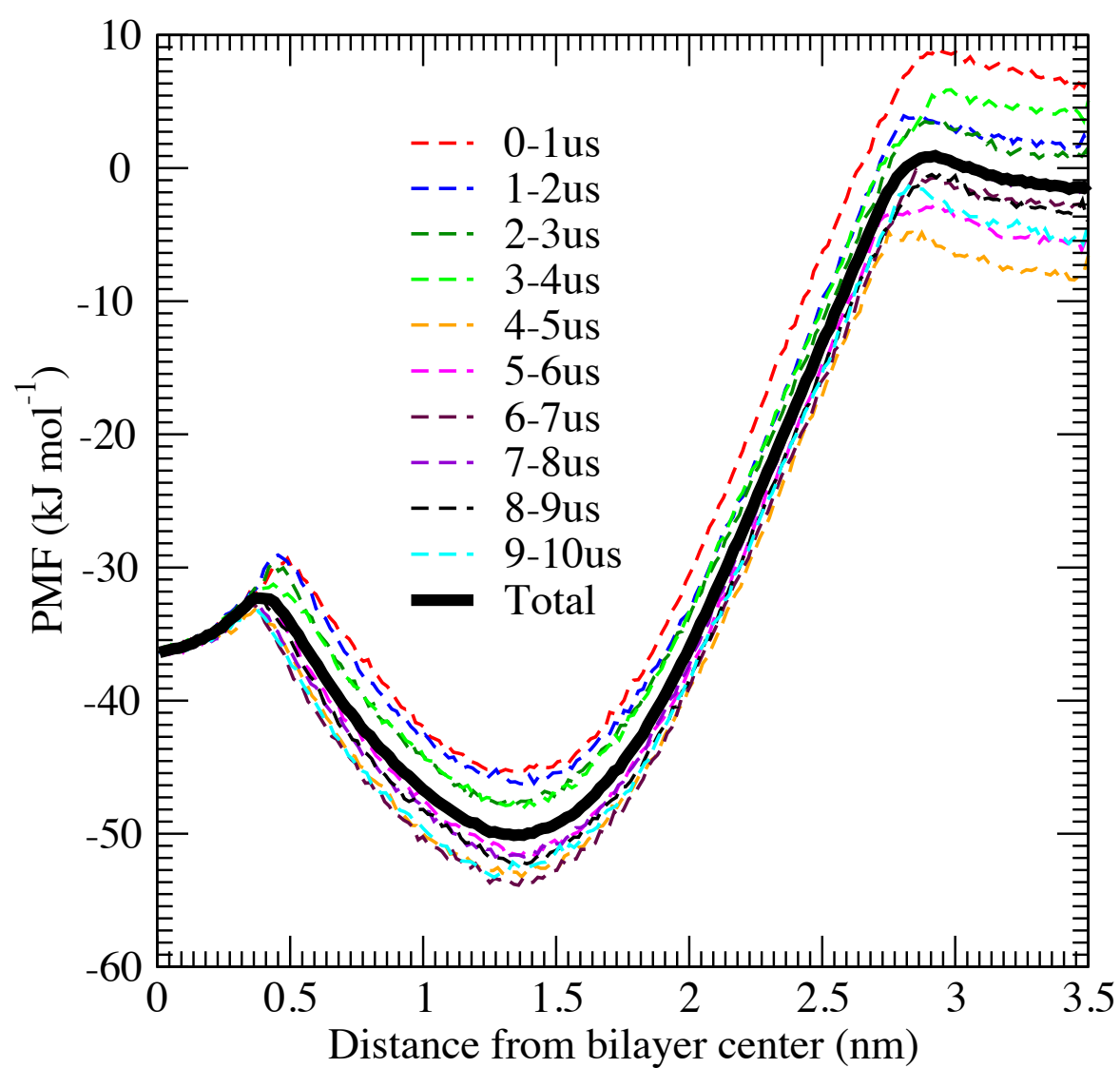

Supplement: S1 Fig — In addition to the plot reported in the main manuscript, the error in the calculated PMF was obtained through block averaging. Thus, independent PMFs were obtained from trajectory blocks of 1 μs (dash lines). The total PMF was obtained from total averaging (solid black line). (PDF) [file pcbi.1005972.s002.pdf]

A

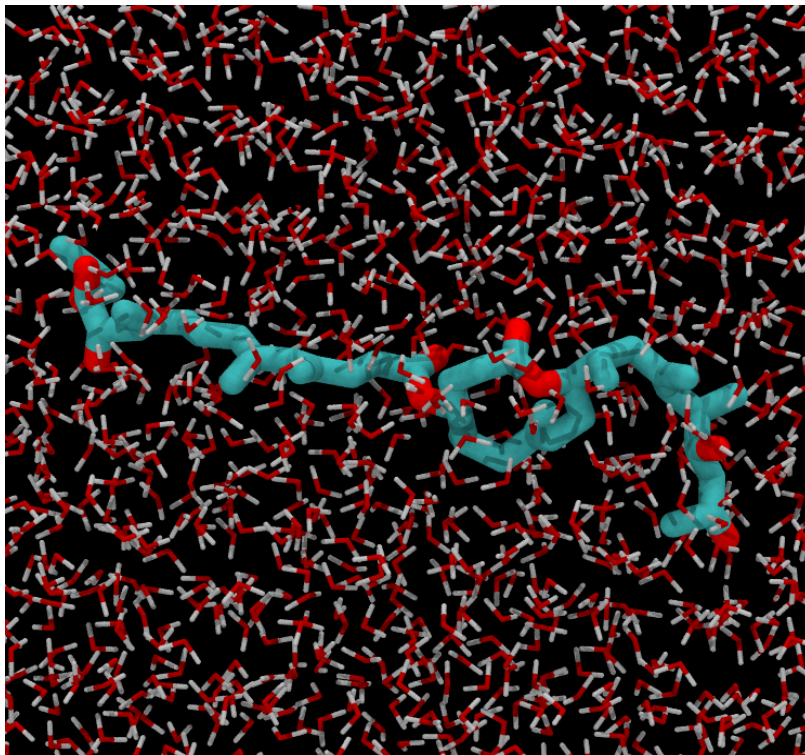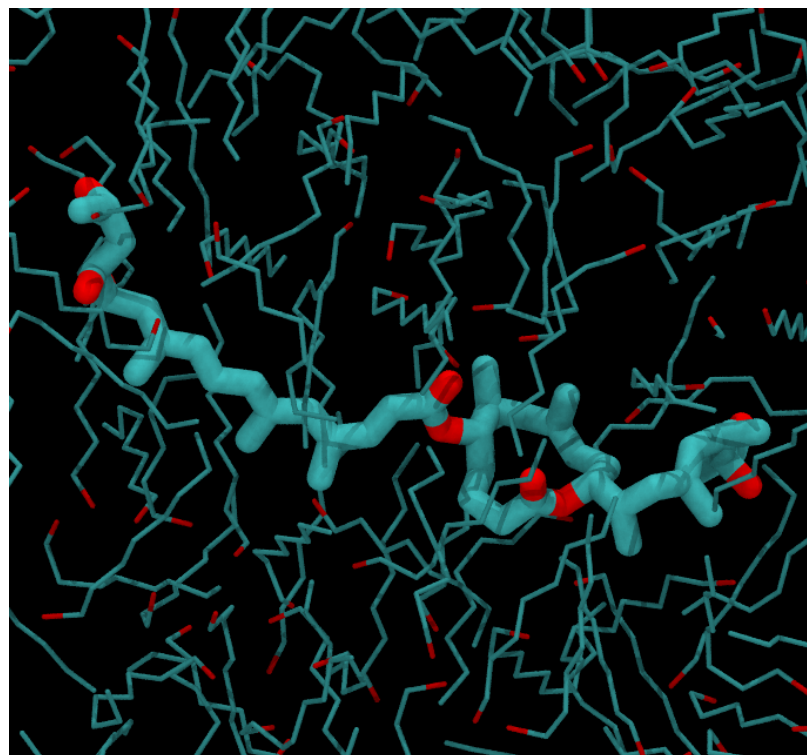

B

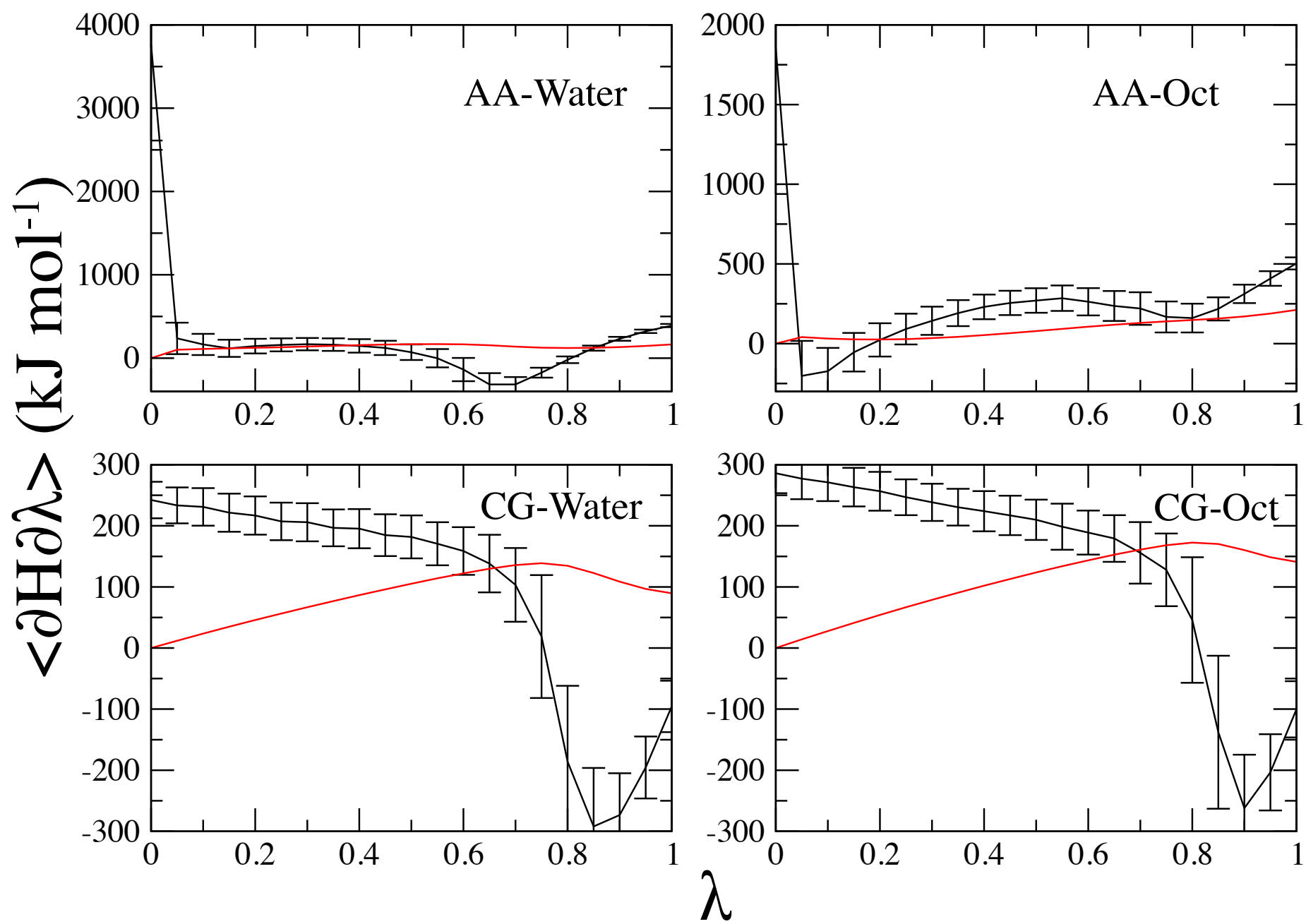

Supplement: S2 Fig — A) Snapshots of mycolactone in either water (left panel) and octanol (right panel) simulation boxes. B) Running derivatives for thermodynamic integration of mycolactone atomistic representation (top panels) and coarse-grained representation (bottom panels). Red lines correspond to the integral. (PDF) [file pcbi.1005972.s003.pdf]

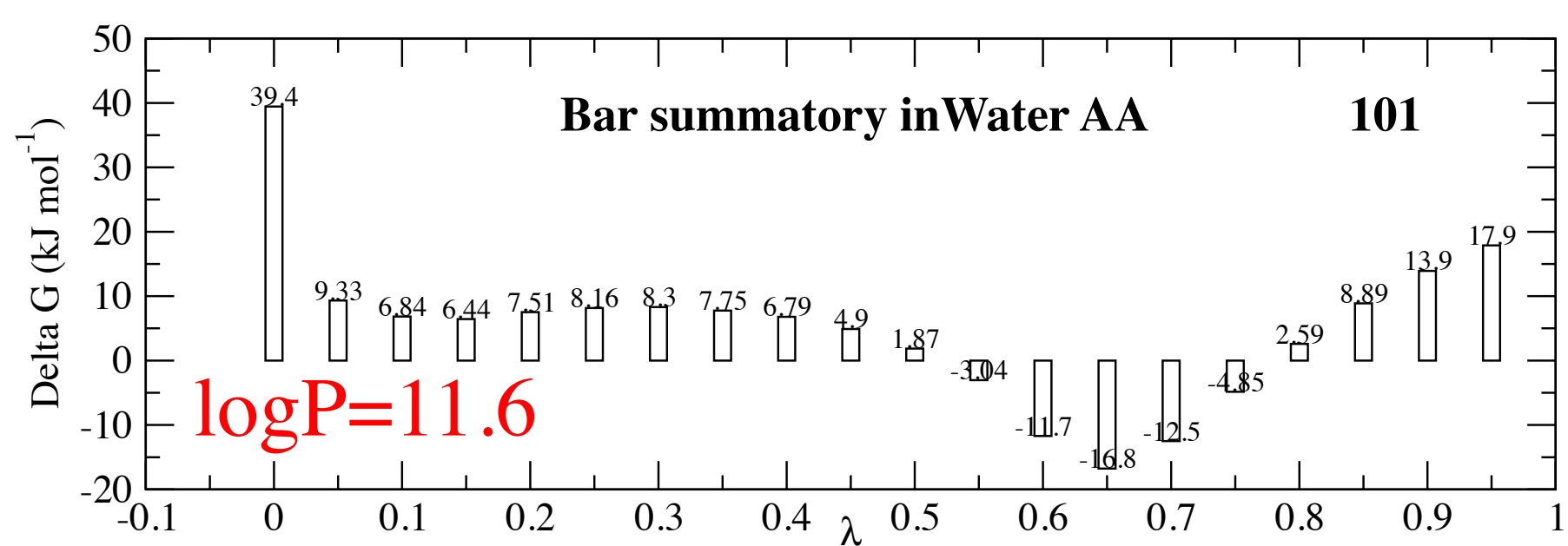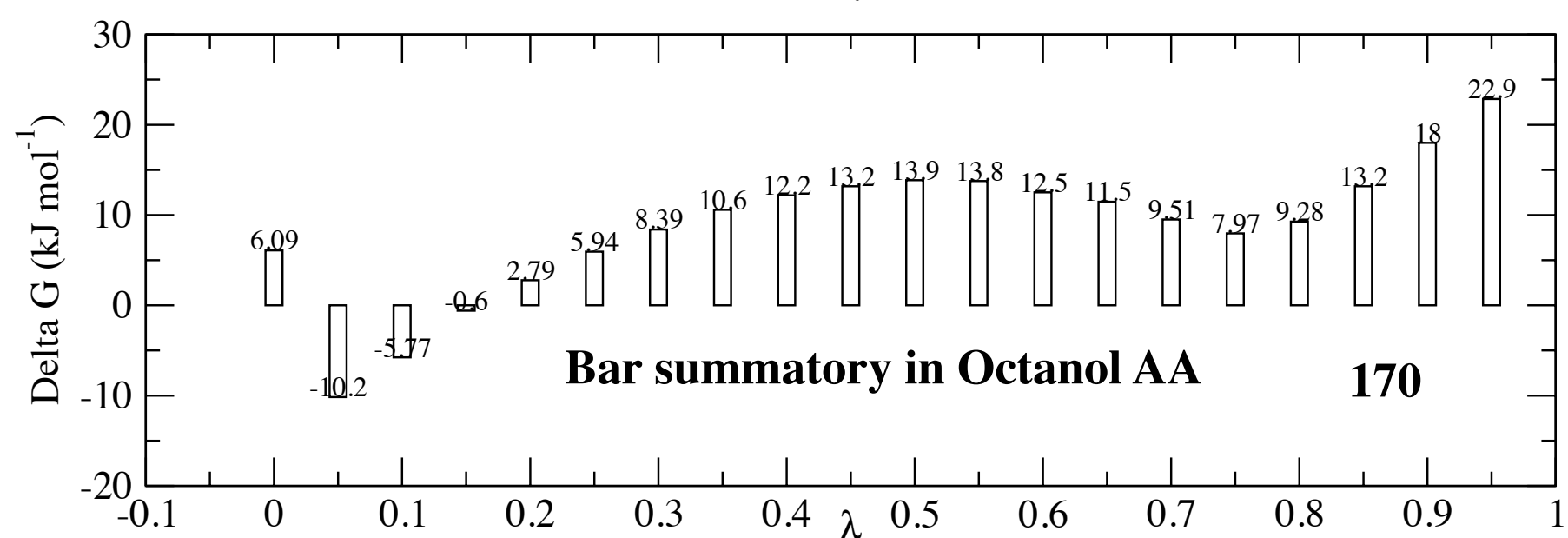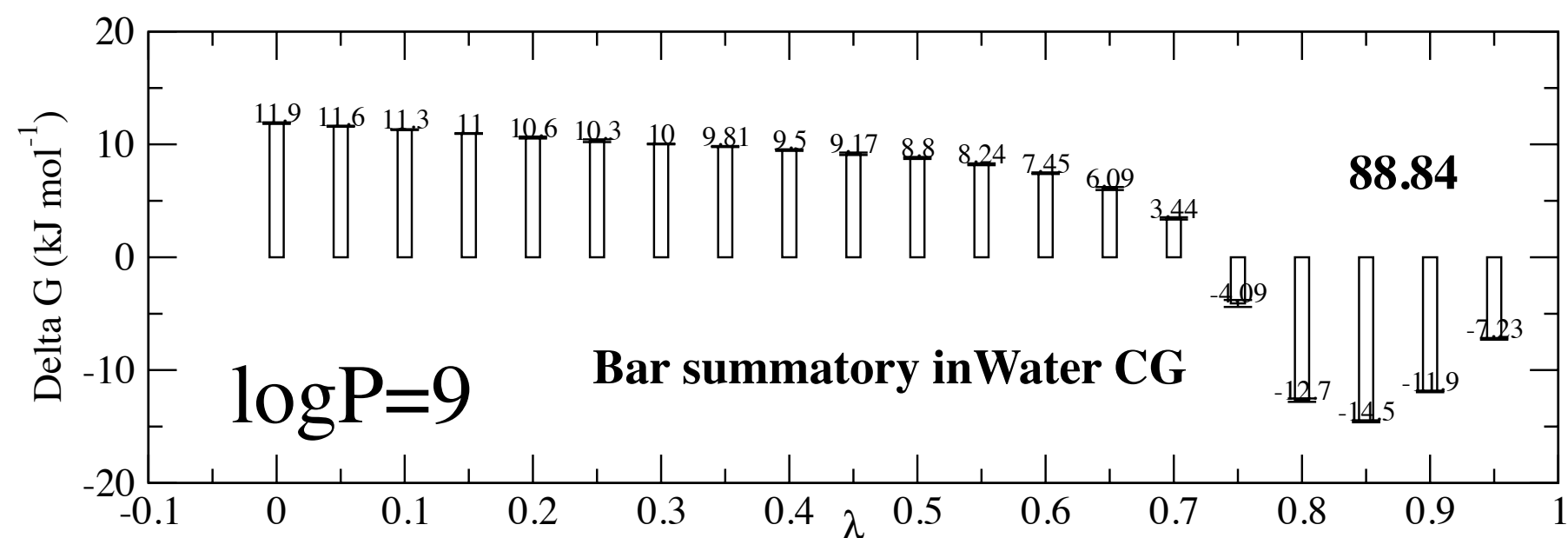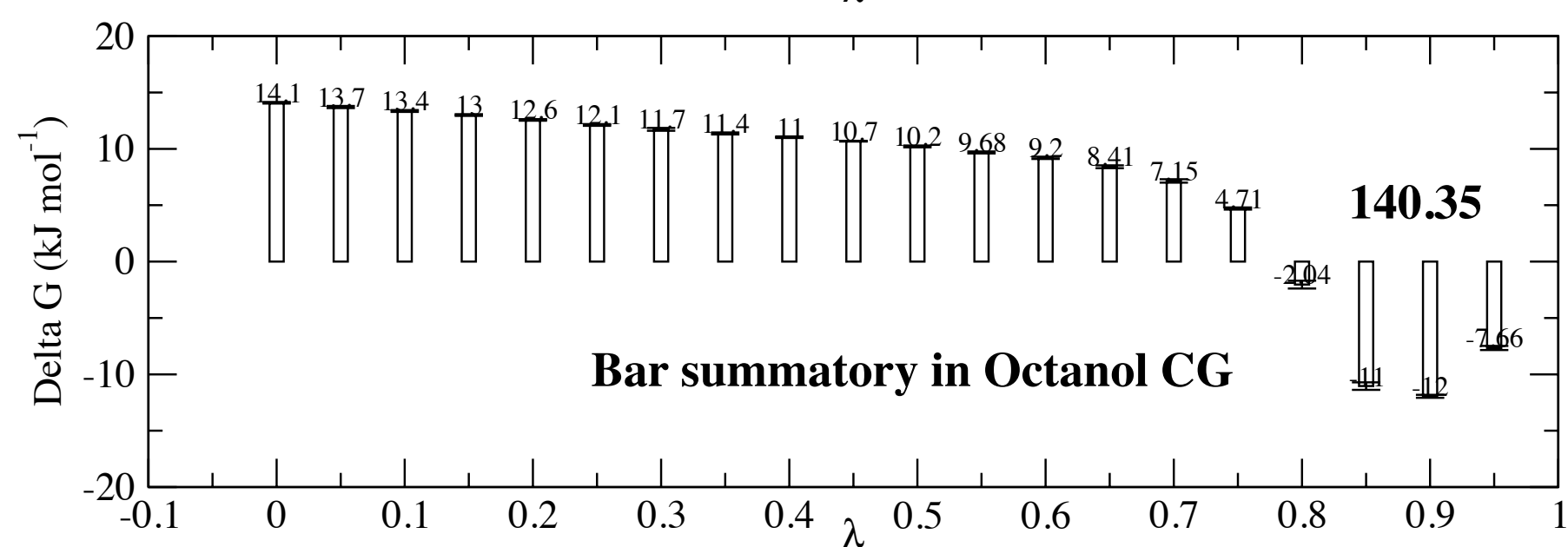

Supplement: S3 Fig — The λ dependency was computed using the g_bar tool as implemented in GROMACS and logP values (red = AA, black = CG) were obtained using equation referenced in the main manuscript. (PDF) [file pcbi.1005972.s004.pdf]
