# Supplementary material for: Membrane perturbing properties of toxin mycolactone from Mycobacterium ulcerans
Source: PLoS Comput Biol. 2018 Feb 5;14(2):e1005972. doi: 10.1371/journal.pcbi.1005972 (PMC5814095; doi:10.1371/journal.pcbi.1005972)
Supplement: S1 Text — (PDF) [file pcbi.1005972.s005.pdf]

Membrane association constant and cellular distribution

We seek to compare the non-specific association of mycolactone with membranes to its specific association with a cytosolic target such as WASP. As stated in the paper the association constant for the membrane is calculated from the PMF according to:

$$K_{\mathrm{ns}} = \frac{[P]_{\mathrm{mem}}}{[P]_{\mathrm{aq}}} = \frac{(c^{\ominus})^{1/3} \int_{mem} \exp[-\beta W(z)]\, dz}{\int \delta(z - z^{*}) \exp[-\beta W(z)]\, dz}$$

In comparison, a specific association constant with WASP is

$$K_{\mathrm{s}} = \frac{[PW]}{[P]_{aq}[W]}$$

where $[PW]$ and $[W]$ are the concentrations of mycolactone-bound and free WASP, respectively. To properly account for standard states [1], each concentration should be multiplied by an activity coefficient (generally assumed to be 1 in the limit of infinite dilution), and divided by the standard state concentration (1 M, or 1 molecule/1.66 nm^3 for solutes in solution). In the absence of an accepted standard state definition for membranes, one can assume that two of the three dimensions from bulk remain, yielding 1 molecule/1.4 nm^2. This leaves the remaining one degree of freedom in the numerator, $(c^{\ominus})^{1/3}$ = 1/1.18 nm, consistent with previous analysis [2].

For the relative concentration of the toxin in membranes and bound to WASP, one needs to set the available membrane and cytosolic binding sites on the same footing. Here we consider the number of competing sites per cell,

$$K_{\mathrm{ns}} = \frac{[P]_{\mathrm{mem}}}{[P]_{\mathrm{aq}}[L]}$$

where $[L]$ is the concentration of membrane (lipid) binding sites per cell. Measuring $[W]$ as the number of WASP molecules per cell, we have:

$$\frac{[P]_{\mathrm{mem}}}{[PW]} = \frac{K_{\mathrm{ns}}[L]}{K_{\mathrm{s}}[W]}$$

Note that K_{ns} and K_S are both unitless as they should be [1], with $[P]_{mem}$ and [L] having units of molecules/$length^2$, while [PS] and [W] have units of molecules/$length^3$ (M).

For a model neutrophil, the estimated WASP concentration is 9 μM [3] and the radius is 4.15 μm [4]. It is estimated that only ~60% [5] of the cellular volume (299.4 μm^3) is available cytosolic space, leaving 179.6 μm^3. Thus, $[W]$ = $N_a \cdot (9 \times 10^{-6}$ M) $(1.796 \times 10^{-13}$ L) = 9.74×10^5 molecules/cell.

Since each mycolactone molecule interacts with ~6 lipids and the area per lipid is ~0.63 nm^2, we can estimate each mycolactone binding site is 3.78×10^{-6} μm^2. Similar to the limited cytosolic space, ~40% of the total plasma membrane (216.42 μm^2) is taken up by protein and glycolipids, leaving 129.9 μm^2. Thus, the available membrane binding sites can be approximated as $[L]$ = (129.9 μm^2/cell) (1 site/3.78×10^{-6} μm^2) = 3.43×10^7 sites/cell.

Thus,

$$\frac{[P]_{\text{mem}}}{[PW]} = \frac{K_{\text{ns}}[L]}{K_{\text{s}}[W]} = \frac{(3.92 \text{ x } 10^{7})(3.43 \text{ x } 10^{7})}{(5.8 \text{ x } 10^{6})(9.74 \text{ x } 10^{5})} = 238$$

suggesting the ratio of toxin molecules bound to the membrane versus WASP is 238:1. Although this seems high given the known association of mycolactone with WASP, this estimate is approximate for several reasons. First, because we are using a model membrane composed of pure DPPC and modeled at the MARTINI CG level. The affinity for a real plasma membrane may be less than what is reported here. Second, this model takes no account of local concentrations of WASP which is known to associate with membranes and thus to have an enhanced concentration close to the internal membrane leaflet. And third, this estimate assumes an even distribution of mycolactone throughout the membrane in spite of our findings suggesting it preferentially localizes at membrane interfaces. Despite these limitations, our results clearly demonstrate a strong association between mycolactone and membranes, and thus the likely role of membrane trafficking in its pathogenicity.

References:

1. Gilson MK, Given JA, Bush BL, McCammon JA. The statistical-thermodynamic basis for computation of binding affinities: a critical review. Biophysical journal. 1997;72(3):1047-69. doi: 10.1016/S0006-3495(97)78756-3. PubMed PMID: 9138555; PubMed Central PMCID: PMC1184492.
2. Zhang L, Yethiraj A, Cui Q. Free Energy Calculations for the Peripheral Binding of Proteins/Peptides to an Anionic Membrane. 1. Implicit Membrane Models. Journal of Chemical Theory and Computation. 2014;10(7):2845-59. doi: 10.1021/ct500218p.
3. Higgs HN, Pollard TD. Activation by Cdc42 and PIP(2) of Wiskott-Aldrich syndrome protein (WASp) stimulates actin nucleation by Arp2/3 complex. The Journal of cell biology. 2000;150(6):1311-20. PubMed PMID: 10995437; PubMed Central PMCID: PMC2150692.
4. Rosenbluth MJ, Lam WA, Fletcher DA. Force microscopy of nonadherent cells: a comparison of leukemia cell deformability. Biophysical journal. 2006;90(8):2994-3003. doi: 10.1529/biophysj.105.067496. PubMed PMID: 16443660; PubMed Central PMCID: PMC1414579.
5. Schmid-Schonbein GW, Shih YY, Chien S. Morphometry of human leukocytes. Blood. 1980;56(5):866-75. PubMed PMID: 6775712.
